# Nicotinic Acid Adenine Dinucleotide Phosphate Induces Intracellular Ca^2+^ Signalling and Stimulates Proliferation in Human Cardiac Mesenchymal Stromal Cells

**DOI:** 10.3389/fcell.2022.874043

**Published:** 2022-03-15

**Authors:** Pawan Faris, Claudio Casali, Sharon Negri, Lara Iengo, Marco Biggiogera, Angela Serena Maione, Francesco Moccia

**Affiliations:** ^1^ Laboratory of General Physiology, Department of Biology and Biotechnology “Lazzaro Spallanzani”, University of Pavia, Pavia, Italy; ^2^ Laboratory of Cell Biology and Neurobiology, Department of Biology and Biotechnology “Lazzaro Spallanzani”, University of Pavia, Pavia, Italy; ^3^ Vascular Biology and Regenerative Medicine Unit, Centro Cardiologico Monzino, IRCCS, Milan, Italy

**Keywords:** nicotinic acid adenine dinucleotide phosphate (NAADP), two-pore channels (TPCs), membrane contact sites, store operated Ca^2+^ entry, cardiac mesenchymal stem cells, proliferation

## Abstract

Nicotinic acid adenine dinucleotide phosphate (NAADP) is a newly discovered second messenger that gates two pore channels 1 (TPC1) and 2 (TPC2) to elicit endo-lysosomal (EL) Ca^2+^ release. NAADP-induced lysosomal Ca^2+^ release may be amplified by the endoplasmic reticulum (ER) through the Ca^2+^-induced Ca^2+^ release (CICR) mechanism. NAADP-induced intracellular Ca^2+^ signals were shown to modulate a growing number of functions in the cardiovascular system, but their occurrence and role in cardiac mesenchymal stromal cells (C-MSCs) is still unknown. Herein, we found that exogenous delivery of NAADP-AM induced a robust Ca^2+^ signal that was abolished by disrupting the lysosomal Ca^2+^ store with Gly-Phe β-naphthylamide, nigericin, and bafilomycin A1, and blocking TPC1 and TPC2, that are both expressed at protein level in C-MSCs. Furthermore, NAADP-induced EL Ca^2+^ release resulted in the Ca^2+^-dependent recruitment of ER-embedded InsP_3_Rs and SOCE activation. Transmission electron microscopy revealed clearly visible membrane contact sites between lysosome and ER membranes, which are predicted to provide the sub-cellular framework for lysosomal Ca^2+^ to recruit ER-embedded InsP_3_Rs through CICR. NAADP-induced EL Ca^2+^ mobilization via EL TPC was found to trigger the intracellular Ca^2+^ signals whereby Fetal Bovine Serum (FBS) induces C-MSC proliferation. Furthermore, NAADP-evoked Ca^2+^ release was required to mediate FBS-induced extracellular signal-regulated kinase (ERK), but not Akt, phosphorylation in C-MSCs. These finding support the notion that NAADP-induced TPC activation could be targeted to boost proliferation in C-MSCs and pave the way for future studies assessing whether aberrant NAADP signaling in C-MSCs could be involved in cardiac disorders.

## 1 Introduction

Nicotinic acid adenine dinucleotide phosphate (NAADP) has emerged as a the most powerful (already in the nanomolar concentration range) Ca^2+^-releasing second messenger in mammalian cells (Galione, 2015; Patel, 2015). NAADP elicits an increase in intracellular Ca^2+^ concentration ([Ca^2+^]_i_) by gating a novel family of intracellular Ca^2+^-releasing channels, known as two-pore channels (TPCs), which present two isoforms in mammals (i.e., TPC1 and TPC2) and mobilize endo-lysosomal (EL) Ca^2+^ into the cytosol (Patel, 2015; Galione, 2019; Jin et al., 2020). Jupiter microtubule-associated homolog 2 (JPT2) (Gunaratne et al., 2021) and the RNA-binding protein, Lsm2 (Zhang et al., 2021), serve as auxiliary protein to bind NAADP and thereby contribute to mediate TPC-mediated EL Ca^2+^ release. The Ca^2+^ response to NAADP may remain spatially confined in proximity of EL vesicles (Ruas et al., 2010; Vassileva et al., 2020) or it can be amplified into a regenerative Ca^2+^ wave through the Ca^2+^-dependent recruitment of juxtaposed ryanodine and inositol-1,4,5-trisphosphate (InsP_3_) receptors at membrane contact sites (MCSs) between lysosomes and endoplasmic reticulum (ER) (Kinnear et al., 2004; Davis et al., 2012; Kilpatrick et al., 2013; Penny et al., 2014). Lysosomal Ca^2+^ refilling is impaired by alkalinization of the EL lumen (Ronco et al., 2015), although the mechanisms whereby intraluminal pH recharges EL vesicles with Ca^2+^ remains a controversial issue (Morgan et al., 2011; Garrity et al., 2016; Faris et al., 2018).

NAADP has been recognized as the trigger of the cellular Ca^2+^ response to extracellular stimuli in multiple tissues (Galione, 2015; Patel, 2015), including the cardiovascular system (Fameli et al., 2017; Moccia et al., 2021a; Negri et al., 2021b). NAADP-induced Ca^2+^ release through TPC2 increases the Ca^2+^ content within the sarcoendoplasmic reticulum in ventricular (Macgregor et al., 2007) and atrial myocytes (Collins et al., 2011), both at rest (Macgregor et al., 2007; Collins et al., 2011) and during β-adrenergic receptor stimulation (Macgregor et al., 2007; Collins et al., 2011; Lewis et al., 2012; Capel et al., 2015). Likewise, a flurry of reports showed that NAADP-induced intracellular Ca^2+^ signals elicit contraction in multiple types of vascular smooth muscle cells (VSMCs) (Kinnear et al., 2004; Jiang et al., 2013; Fameli et al., 2014; Trufanov et al., 2019). For instance, NAADP gates TPC2 to promote the Ca^2+^-dependent recruitment of RyR3 and global cytosolic Ca^2+^ waves in pulmonary artery VSMCs stimulated with either endothelin-1 (Kinnear et al., 2004; Jiang et al., 2013) or angiotensin II (Lee et al., 2015). Finally, NAADP may serve as a trigger of the Ca^2+^ response to extracellular stimuli also in vascular endothelial cells (Favia et al., 2014; Zuccolo et al., 2019; Negri et al., 2021a) and circulating endothelial colony forming cells (ECFCs) (Balducci et al., 2021; Moccia et al., 2021b). Aberrant NAADP signalling in cardiac myocytes may result in arrhythmia (Nebel et al., 2013) and ischemia-reperfusion injury (Davidson et al., 2015), whereas it could lead to pulmonary artery hypertension in VSMCs (Jiang et al., 2018; Hu et al., 2021).

Once regarded as mere bystanders of the contractile function effected by neighbouring cardiac myocytes, cardiac mesenchymal stromal cells (C-MSCs) are required to maintain myocardial structure and function and, therefore, to ensure effective cardiac contraction (Brown et al., 2005; Camelliti et al., 2005). C-MSCs contribute to wound healing and fibrotic remodelling after ischemic injury (Jugdutt, 2003; Camelliti et al., 2005) and they have been put forward as a promising cellular substrate to induce cardiac repair (Bagno et al., 2018; Braunwald, 2018). Furthermore, C-MSCs could stimulate cardiac myocytes to undergo proliferation or hypertrophy depending on whether this interaction takes place during embryonic development or in the adult heart (Kakkar and Lee, 2010). Finally, C-MSCs exhibit significant immunomodulatory potential by attenuating the inflammatory response in the infarcted myocardium (Czapla et al., 2016; Diedrichs et al., 2019). In agreement with their contribution to the structural, biochemical and electro-chemical features of the myocardium, C-MSCs are involved in the pathogenic mechanisms of multiple cardiac diseases (Brown et al., 2005; Camelliti et al., 2005). For instance, C-MSCs provide a source of adipocytes (Sommariva et al., 2016; Stadiotti et al., 2017) and support fibrotic remodelling (Maione et al., 2021) in arrhythmogenic cardiomyopathy (ACM), a rare genetic disorder that is featured by fibro-fatty myocardium substitution, malignant arrhythmias, and heart failure and that can lead to sudden death in young individuals (Moccia et al., 2019). It has long been known that an increase in [Ca^2+^]_i_ regulates multiple functions in human MSCs (Moccia et al., 2015; Forostyak et al., 2016; Jiang et al., 2017), including proliferation (Foreman et al., 2006), migration (Peng et al., 2016), gene expression (Kawano et al., 2006), and differentiation (Kawano et al., 2006; Tao et al., 2011). However, it is still unclear whether and how NAADP evokes intracellular Ca^2+^ signals and whether lysosomal-ER MCSs do exist in C-MSCs. This information could be extremely helpful to boost the design of alternative strategies to effectively target C-MSCs in a variety of life-threatening cardiac disorders. In the present investigation, we first provided the evidence that NAADP evokes robust lysosomal Ca^2+^ mobilization, which is amplified into a global increase in [Ca^2+^]_i_ by InsP_3_ receptors (InsP_3_Rs). Transmitted electron microscopy (TEM) then revealed clearly discernible MCSs between lysosomes and ER membrane in C-MSCs. Finally, we found that NAADP-induced Ca^2+^-dependent crosstalk between lysosomes and ER triggers the intracellular Ca^2+^ signals whereby Fetal Bovine Serum (FBS) induces cell proliferation. The role of Ca^2+^ signalling in regulating proliferation and differentiation in MSCs confer these findings the potential to provide the molecular framework for further studies aiming at manipulating C-MSCs for therapeutic purposes.

## 2 Materials and Methods

### 2.1 Ethical Statement

This study complies with the WMA Declaration of Helsinki. The use of human cells from biopsy samples of healthy subjects (cardiomyopathies ruled out) was approved by IEO-CCM IRCCS Ethic Committee (project CCM1072). Written informed consent was obtained from all participants.

### 2.2 C-MSC Isolation and Culture

Cells were obtained from endomyocardial specimens and characterized as previously described (Pilato et al., 2018) and cultured with Iscove’s Modified Dulbecco’s Medium (Thermo Fisher Scientific, MA, United States) supplemented with 20% Fetal Bovine Serum (FBS), 10 ng/ml basic fibroblast growth factor, 10,000 U/ml Penicillin, 10,000 μg/ml Streptomycin, and 0.02 M L-Glutamine.

### 2.3 Solutions

Physiological salt solution (PSS) had the following composition (in mM): 150 NaCl, 6 KCl, 1.5 CaCl_2_, 1 MgCl_2_, 10 Glucose, 10 Hepes. In Ca^2+^-free solution (0Ca^2+^), Ca^2+^ was substituted with 2 mM NaCl, and 0.5 mM EGTA was added. Solutions were titrated to pH 7.4 with NaOH. The osmolality of the extracellular solution, as measured with an osmometer (Wescor 5500, Logan, UT, United States), was 300–310 mmol/kg.

### 2.4 [Ca^2+^]_i_ Measurements and Statistics of Ca^2+^ Signals

C-MSCs were loaded with 2 µM fura-2 acetoxymethyl ester (fura-2/AM; 1 mM stock in dimethyl sulfoxide) in PSS for 30 min at room temperature (RT). The details of the Ca^2+^ recording set-up have been described in Moccia et al. (2021b) and are reported in the Supplementary Material. All the experiments were performed at RT. The amplitude of intracellular Ca^2+^ release in response to each agonist (NAADP or FBS) or drug [Gly-Phe β-naphthylamide (GPN), nigericin, bafilomycin A1, and cyclopiazonic acid (CPA)] was measured as the difference between the ratio at the peak of intracellular Ca^2+^ mobilization and the mean ratio of 1 min baseline before the peak. Pooled data are given as mean ± SE and statistical significance (*p* < 0.05) was evaluated by the Student’s t-test for unpaired observations or one-way Anova analysis followed by the post-hoc Dunnett’s test as appropriate (Negri et al., 2021a; Remigante et al., 2021). Data relative to Ca^2+^ signals are presented as mean ± SE, while the number of cells analysed is indicated in the corresponding bar histograms.

### 2.5 mRNA Extraction and qRT-PCR Assay

Cell cultures were lysed in RL lysis buffer (Norgen Biotek Corp., Thorold, ON, Canada). RNA was isolated from cells by using a Total RNA Purification kit (Norgen Biotek Corp., Thorold, ON, Canada). The quantification of the isolated RNA was determined by NanoDrop spectrophotometer (ND-1000, EuroClone, Milan, Italy). Reverse transcription was conducted with SuperScript III (Invitrogen, Carlsbad, CA, United States) following the manufacturer’s instructions. qRT-PCR was performed with the use of the iQTM SYBR Green Super Mix (Bio-Rad Laboratories, Hercules, CA, United States) and specific primers (reported in Table 1). All reactions were performed in a 96-well format with the 7900HT Fast Real-Time PCR System (Thermo Fisher Scientific, MA, United States). The relative quantities of specific mRNA were obtained with the use of the comparative Ct method and were normalized to the housekeeping gene glyceraldehyde 3-phosphate dehydrogenase (GAPDH) (Maione et al., 2021; Zuccolini et al., 2022). The expression of each target gene was assessed in triplicate (Ferrera et al., 2021; Maione et al., 2021).

**TABLE 1:** Primer sequences 5′-3′.

Gene	Forward primer	Reverse primer
*TPC1*	GAG​TTT​GGA​TGA​CGA​CGT​GC	GAG​TCG​TGG​ATG​GCA​TAG​CT
*TPC2*	CTT​ACC​GCA​GCA​TCC​AAG​TC	GTA​AAG​CCA​CAT​CGA​GCT​GG
*GAPDH*	ATG​TTC​GTC​ATG​GGT​GTG​AA	GTC​TTC​TGG​GTG​GCA​GTG​AT

### 2.6 Protein Extraction and Western Blot Analysis

C-MSCs were lysed in cell lysis buffer (Cell Signalling Technology, Danvers, MA, United States) supplemented with protease and phosphatase inhibitor cocktails (Sigma-Aldrich, Saint Louis, MO, United States). Total protein extracts were subjected to SDS-PAGE and transferred onto a nitrocellulose membrane (Bio-Rad, CA, United States). The membranes were blocked for 1 h at room temperature in 5% non-fat dry milk in Wash Buffer (Tris Buffer Sulfate, 0.1% Tween-20) and then incubated O/N at 4°C with the appropriate primary antibodies (reported in Table 2). The membranes were incubated with peroxidase-conjugated secondary antibodies (GE Healthcare, Chicago, IL, United States) for 1 h. Signals were visualized using the LiteUP Western Blot Chemiluminescent Substrate (EuroClone, Milan, Italy). Images were acquired with the ChemiDocTM MP Imaging System (Bio-Rad, CA, United States), and densitometric analysis of membranes was performed using the ImageJ software (National Institutes of Health, Bethesda, MD, United States). C-MSC proteins were normalized according to glyceraldehyde 3-phosphate dehydrogenase (GAPDH) signal.

**TABLE 2:** Primary antibodies.

Protein	Clonality/Code	Source/Isotype	Company	Dilution
TPCN1	Polyclonal, SAB2104213	Rabbit	Sigma-Aldrich	1:1,000
TPC2	Polyclonal, ab119915	Rabbit	Abcam	1:1,000
phospho-ERK1/2	Monoclonal, #4370	Rabbit IgG	Cell Signaling	1:1,000
ERK1/2	Polyclonal, #9102	Rabbit	Cell Signaling	1:1,000
phospho-AKT	Monoclonal, #4056	Rabbit IgG	Cell Signaling	1:1,000
AKT	Polyclonal, #9272	Rabbit	Cell Signaling	1:1,000
GAPDH	Polyclonal, sc-25778	Rabbit	Santa Cruz	1:1,000

### 2.7 Transmission Electron Microscopy

For transmission electron microscopy (TEM) analysis, following trypsinization cells were centrifuged at 800 rpm for 5 min and then fixed with 2.5% glutaraldehyde in culture medium, for 2 h at RT (Carriero et al., 2021). The cell pellet was then rinsed in PBS overnight, post-fixed in 1% aqueous OsO4 for 3 h at room temperature and rinsed in H2O. Cells were pre-embedded in 2% agarose in water, dehydrated in acetone and then embedded in epoxy resin (Electron Microscopy Sciences, EM-bed812). Ultrathin sections (60–80 nm) were cut on a Reichert OM-U3 ultramicrotome, collected on nickel grids and then stained with uranyl acetate and lead citrate. The specimens were observed with a JEM 1200 EX II (JEOL, Peabody, MA, United States) electron microscope operating at 100 kV and equipped with a MegaView G2 CCD camera (Olympus OSIS, Tokyo, Japan).

### 2.8 Cell Proliferation

C-MSCs were plated in 6-well plates (100,000 cells/well) and serum starved for 4 h. Cells were then stimulated with 20% FBS in the absence (Ctrl) or presence of 100 µM of NED-19, a selective TPC blocker (Galione, 2015; Jin et al., 2020). 24 and 48 h after stimulation with FBS, the medium was removed, cells detached from the plates, and counted.

### 2.9 Flow Cytometry

To evaluate whether blocking TPCs with NED-19 was able to induce apoptosis in C-MSCs, Annexin V Alexa Fluor™ 488 Dye (Thermo Fisher Scientific, MA, United States) has been used, according to the manufacturer’s instructions. Briefly, cells were detached using TrypLE™ Select Enzyme (Thermo Fisher Scientific, MA, United States) and incubated with Annexin V Alexa Fluor™ 488 Dye for 15 min at RT. The fluorescence emission at 530 nm corresponding to apoptotic cells has been measured using flow cytometry (Gallios, Beckman Coulter, Brea, CA, United States).

## 3 Results

### 3.1 Nicotinic Acid Adenine Dinucleotide Phosphate Induces Intracellular Ca^2+^ Signals by Mobilizing Lysosomal Ca^2+^ in Cardiac Mesenchymal Stromal Cells

**FIGURE 1:**
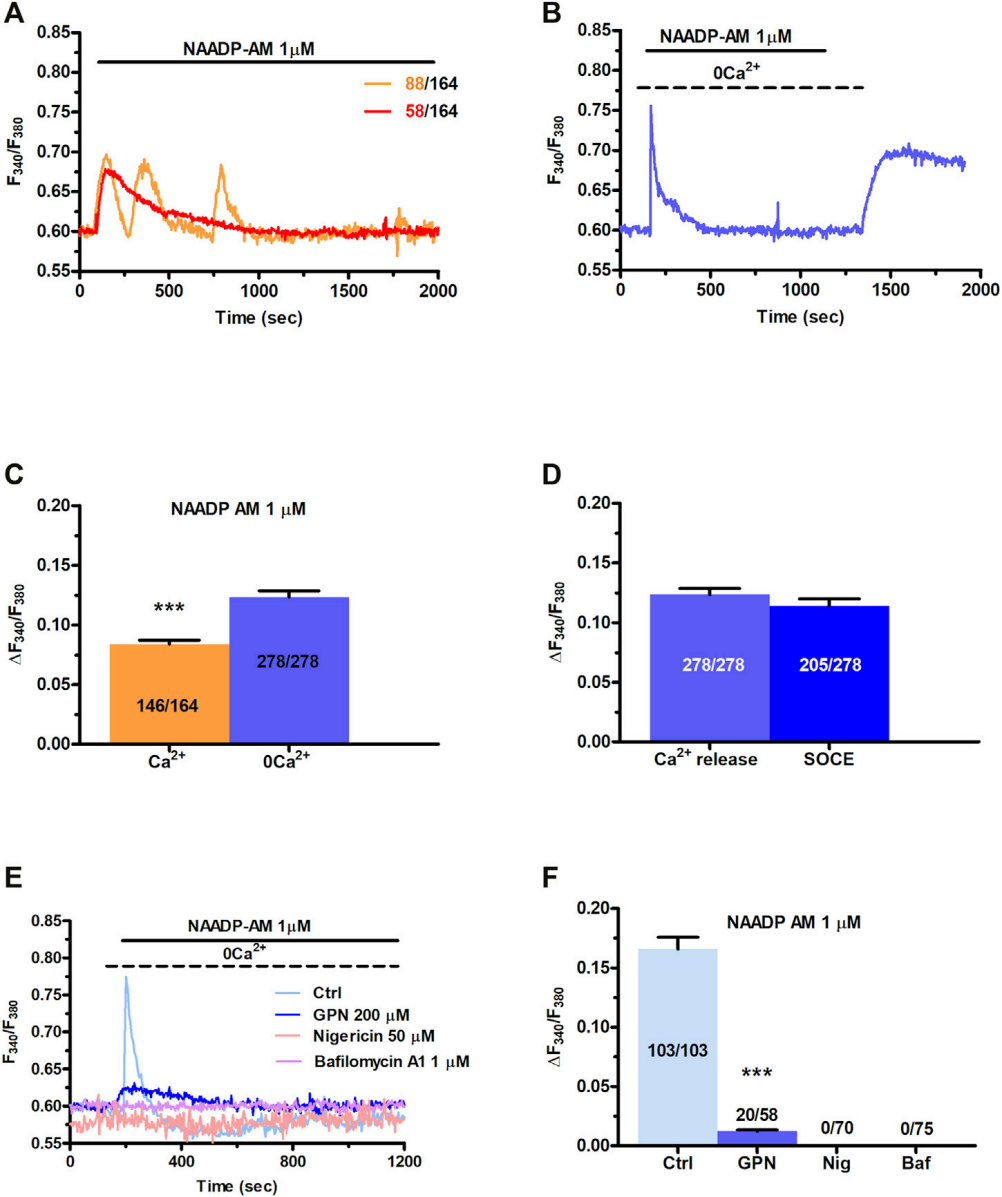
NAADP induces intracellular Ca^2+^ signals by mobilizing lysosomal Ca^2+^ in C-MSCs. **(A)** Exogenous administration of NAADP-AM (1 µM) indues either intracellular Ca^2+^ oscillations or a transient increase in [Ca^2+^]_i_. **(B)** In absence of external Ca^2+^ (0Ca^2+^), NAADP-AM (1 µM) induced only a transient increase in [Ca^2+^]_i_, whereas subsequent restitution of extracellular Ca^2+^ after the full recovery of [Ca^2+^]_i_ to the baseline resulted in a second Ca^2+^ signal that was due to extracellular Ca^2+^ entry. **(C)** Mean ± SE of the amplitude of the peak Ca^2+^ response to NAADP in the presence and absence of extracellular Ca^2+^. Student’s t-test: ***p < 0.001. **(D)** Mean ± SE of the amplitude of NAADP-induced intracellular Ca^2+^ release and SOCE. **(E)** Disrupting the lysosomal Ca^2+^ store with GPN (200 μM, 30 min), nigericin (50 μM, 30 min) or bafilomycin A1 (1 μM, 30 min) severely affected the intracellular Ca^2+^ response to NAADP-AM. **(F)** Mean ± SE of the amplitude of the peak Ca^2+^ response to NAADP-AM in the absence and in the presence of GPN, nigericin (Nig), or bafilomycin A1 (Baf). One-Way Anova followed by the post-hoc Dunnett’s test: ***p < 0.001.

In order to assess whether they are endowed with a NAADP-sensitive Ca^2+^ store, C-MSCs were loaded with Fura-2/AM (2 µM), a Ca^2+^ sensitive fluorophore, as shown elsewhere (Maione et al., 2020a). Human MSCs may exhibit spontaneous oscillations in [Ca^2+^]_i_ (Kawano et al., 2002; Kawano et al., 2003; Kawano et al., 2006). Consistently, a fraction of C-MSCs (≈56.4%) exhibited a few (1-4) Ca^2+^ spikes in the absence of extracellular stimulation (Supplementary Figure S1). These cells were, therefore, discarded from subsequent analysis as shown elsewhere (Zuccolo et al., 2017; Zuccolo et al., 2019), since the spontaneous, unpredictable Ca^2+^ activity could mask or even prevent (in case of transient depletion of endogenous target organelle) the Ca^2+^ response to NAADP. We then assessed whether NAADP-AM, a membrane-permeable analogue of NAADP (Macgregor et al., 2007; Brailoiu et al., 2010), was able to increase the [Ca^2+^]_i_ in C-MSCs. NAADP-AM (1 µM) evoked a short train of intracellular Ca^2+^ oscillations that declined ≈25 min after their onset in the presence of extracellular Ca^2+^ in 88 out of 164 cells (53.6%) (Figure 1A). In 58 out of 164 cells (35.4%), NAADP-AM (1 µM) induced a transient increase in [Ca^2+^]_i_ that lasted ≈800 s returned to the baseline in the continuous presence of the agonist (Figure 1A). Eighteen cells (11%) were not responsive to NAADP-AM (1 µM). Under 0Ca^2+^ conditions, NAADP-AM (1 µM) induced only a transient increase in [Ca^2+^]_i_ that was not followed by additional Ca^2+^ spikes (Figure 1B). Intriguingly, the duration of the elevation in [Ca^2+^]_i_ was significantly shorter, i.e., ≈280 s, while the peak amplitude was higher (Figure 1C), as compared to the Ca^2+^ transient recorded in the presence of extracellular Ca^2+^. The subsequent restitution of extracellular Ca^2+^ after the full recovery of [Ca^2+^]_i_ to the baseline resulted in a second Ca^2+^ signal that was due to extracellular Ca^2+^ entry (Figure 1B). NAADP-AM was removed from the perfusate 100 s before re-addition of extracellular Ca^2+^ (Figure 1B), which suggests that the Ca^2+^ entry pathway recruited downstream of NAADP-AM-induced Ca^2+^ release is provided by store-operated Ca^2+^ entry (SOCE), as more widely discussed below (Yamazaki et al., 2007; Sanchez-Hernandez et al., 2010; Negri et al., 2020). The statistical analysis of the two distinct components of the Ca^2+^ response to NAADP-AM (i.e., endogenous Ca^2+^ release and SOCE) is presented in Figure 1D. NAADP is recognized as a mobilizer of the lysosomal Ca^2+^ pool (Galione, 2015; Patel, 2015). Control experiments confirmed that adding back extracellular Ca^2+^ after 700 s exposure to 0Ca^2+^ conditions did not increase the in C-MSCs (Supplementary Figure S2). In accord, NAADP-AM-evoked intracellular Ca^2+^ release was significantly (*p* < 0.001) reduced by discharging the lysosomal Ca^2+^ store with the lysosomotropic compound, dipeptide glycyl-l-phenylalanine 2-naphthylamide (GPN; 200 μM, 30 min) (Kilpatrick et al., 2013; Yuan et al., 2021) (Figures 1E,F). Of note, GPN has recently been reaffirmed as a reliable pharmacological tool to mobilize lysosomal Ca^2+^ (Yuan et al., 2021). Furthermore, NAADP-AM-evoked endogenous Ca^2+^ mobilization was abolished by collapsing the lysosomal H^+^ gradient that maintains lysosomal Ca^2+^ refilling with the H^+^/K^+^ ionophore, nigericin (50 μM, 30 min) (Figures 1E,F), or with the v-ATPase inhibitor, bafilomycin A1 (1 μM, 30 min) (Figures 1E,F) (Morgan et al., 2011; Ronco et al., 2015; Faris et al., 2019; Yuan et al., 2021). Supplementary Figure S3 shows that GPN (200 µM), nigericin (50 µM), and bafilomycin A1 (1 µM) induced a remarkable reduction in Lysotracker Red fluorescence, thereby confirming that all of these drugs target lysosomal Ca^2+^ (Pandey et al., 2009; Faris et al., 2019; Yuan et al., 2021). In accord with these observations, ammonium chloride (NH_4_Cl), which disrupts the lysosomal Ca^2+^ pool by inducing intraluminal alkalinization (Christensen et al., 2002), also reduced Lysotracker Red Fluorescence and impaired NAADP-AM-evoked intracellular Ca^2+^ mobilization (Supplementary Figure S4).

Overall, these findings provide the first evidence that NAADP may induce lysosomal Ca^2+^ release followed by extracellular Ca^2+^ entry in C-MSCs.

### 3.2 Nicotinic Acid Adenine Dinucleotide Phosphate-Induced Intracellular Ca^2+^ Release is Mediated by TPCs in Cardiac Mesenchymal Stromal Cells

**FIGURE 2:**
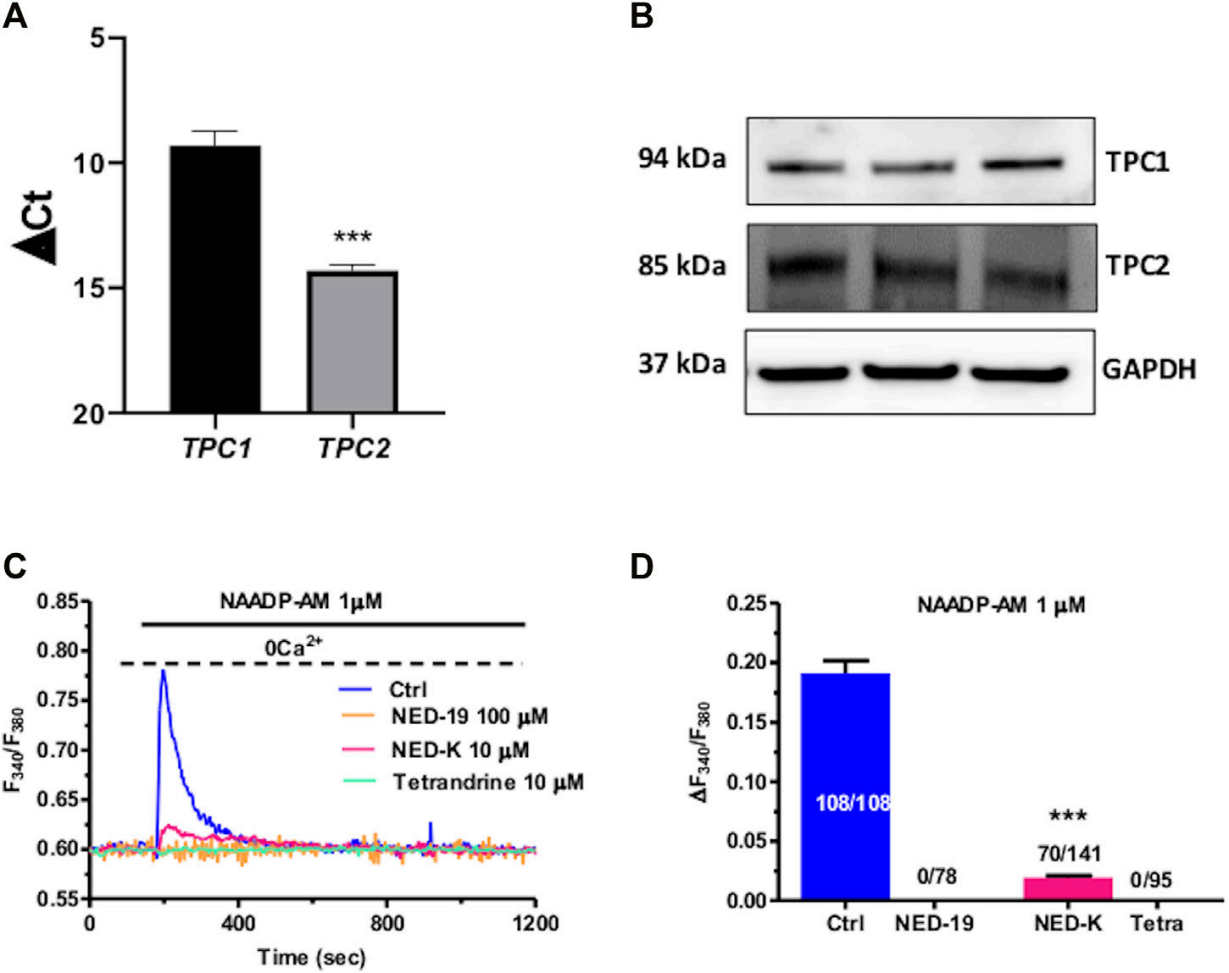
Two-pore channels (TPCs) mediate NAADP-induced lysosomal Ca^2+^ release in C-MSCs. **(A)** TPC1 and TPC2 gene expression in total RNA extracts of C-MSCs. qRT-PCR data are shown as transcript abundance (genes threshold cycles [Ct] with respect to the house-keeping gene GAPDH). *n* = 4/group. Student’s t-test: ***p < 0.001. **(B)** Western Blot analysis of TPC1 and TPC2 proteins in total cellular extracts. *n* = 3/group. **(C)** The Ca^2+^ response to NAADP-AM was suppressed by incubating the cells with the following TPC inhibitors: NED-19 (100 μM, 30 min), NED-K (10 μM, 30 min), and tetrandrine (10 μM, 30 min). **(D)** Mean ± SE of the amplitude of the peak Ca^2+^ response to NAADP in the absence (Ctrl) and in the presence of NED-19, NED-K and tetrandrine (Tetra). Student’s t-test: ***p < 0.001.

TPCs mediate NAADP-induced intracellular Ca^2+^ release throughout the phylogenetic tree (Patel, 2015; Galione, 2019; Jin et al., 2020), including the cardiovascular system (Moccia et al., 2021a; Negri et al., 2021b). In accord, qRT-PCR analysis showed that both TPC1 and TPC2 transcripts are expressed in C-MSCs, although TPC1 mRNA is slightly more abundant (Figure 2A). Negative controls were performed by omitting reverse transcriptase from the reaction (not shown) (Faris et al., 2019). Immunoblotting confirmed that TPC1 and TPC2 are also expressed at protein level. Two single bands of, respectively, 94 and 85 kDa were found for TPC1 and TPC2 proteins (Figure 2B). C-MSCs are not amenable for lipofectamine-mediated transfection of selective small interfering RNAs (Maione, Sommariva, and Pompilio, unpublished results), which is the strategy we have recently employed to downregulate TPC1 expression in different cellular models (Faris et al., 2019; Moccia et al., 2021b). Therefore, we probed the effect of NED-19, a selective TPC inhibitor (Galione, 2015; Jin et al., 2020), which has been widely employed to inhibit NAADP-dependent TPC activation throughout the cardiovascular system (Macgregor et al., 2007; Jiang et al., 2013; Hu et al., 2021; Moccia et al., 2021a; Negri et al., 2021a). As predicted, NED-19 (100 μM, 30 min) fully suppressed NAADP-AM-evoked intracellular Ca^2+^ mobilization (Figures 2C,D). Likewise, NED-K (10 μM, 30 min), a chemically modified analogue of NED-19 that has recently been shown to selectively inhibit TPC1 (Davidson et al., 2015), and tetrandrine (10 μM, 30 min), a traditional Chinese herbal remedy that block both TPC1 and TPC2 (Sakurai et al., 2015; Moccia et al., 2021a), respectively, inhibited (*p* < 0.001) and abrogated NAADP-AM-evoked intracellular Ca^2+^ release (Figures 2C,D). In aggregate, these data demonstrate that NAADP stimulates TPCs to mobilize lysosomal Ca^2+^ in c-MSCs.

### 3.3 InsP_3_Rs at MCSs are Activated Downstream of NAADP-AM-Induced Intracellular Ca^2+^ Release in Cardiac Mesenchymal Stromal Cells

**FIGURE 3:**
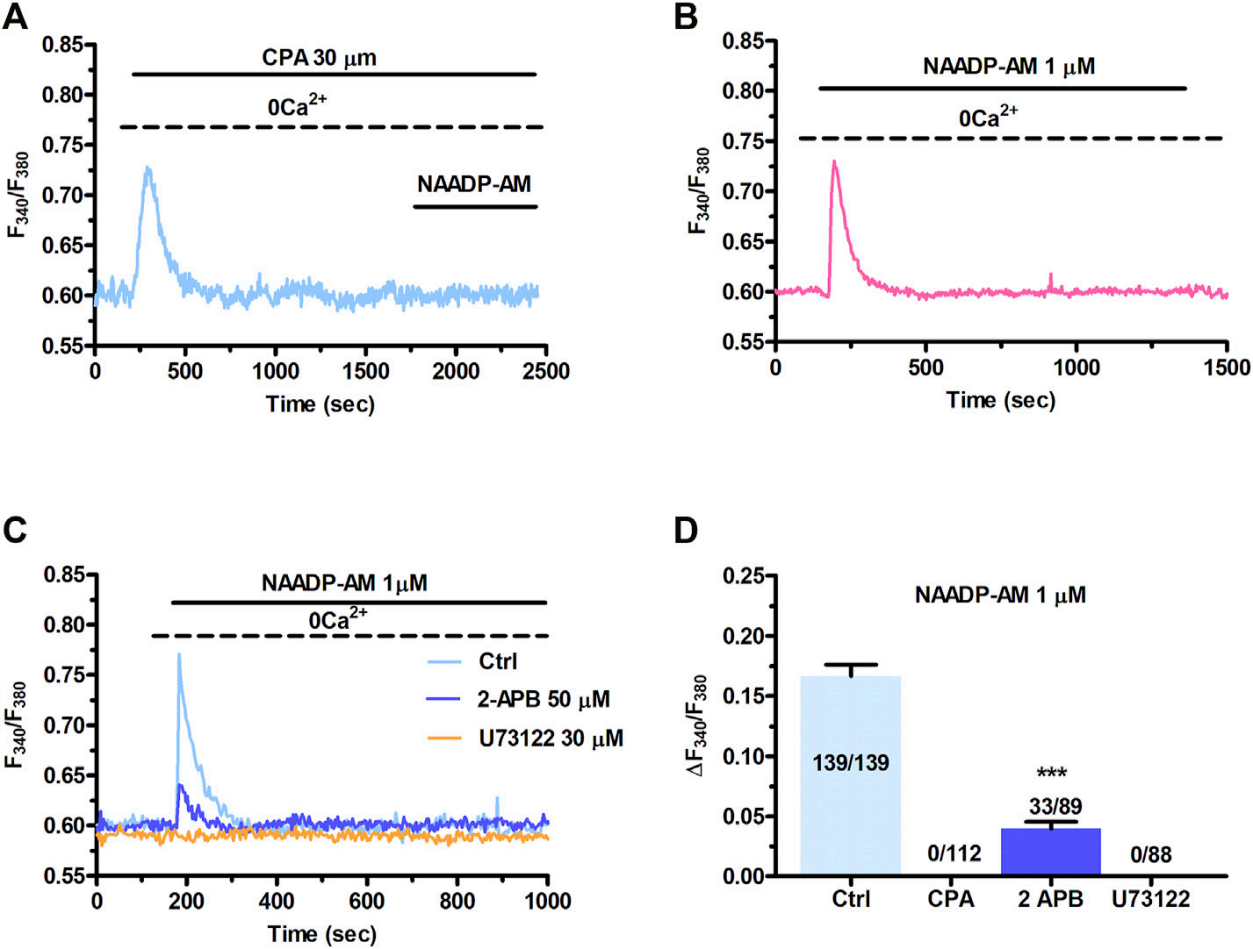
InsP_3_Rs support the Ca^2+^ response to NAADP. **(A)** Administration of NAADP-AM (1 µM) after the pharmacological depletion of the ER Ca^2+^ pool with CPA (30 μm, 30 min) failed to induce intracellular Ca^2+^ release. CPA induced a transient increase in [Ca^2+^]_i_ that reflects passive ER Ca^2+^ leakage from the ER. **(B)** The intracellular Ca^2+^ transient evoked by NAADP-AM (1 µM) in the absence of extracellular Ca^2+^ (0Ca^2+^) under control conditions. **(C)** The intracellular Ca^2+^ release evoked by NAADP-AM (1 µM) under control (Ctrl) conditions was severely affected by blocking InsP_3_Rs with 2-APB (50 μm, 30 min) or inhibiting PLC activity with U73122 (10 μm, 10 min). **(D)** Mean ± SE of the amplitude of the peak intracellular Ca^2+^ response to NAADP-AM under the designated treatments. Student’s t-test: ***p < 0.001.

**FIGURE 4:**
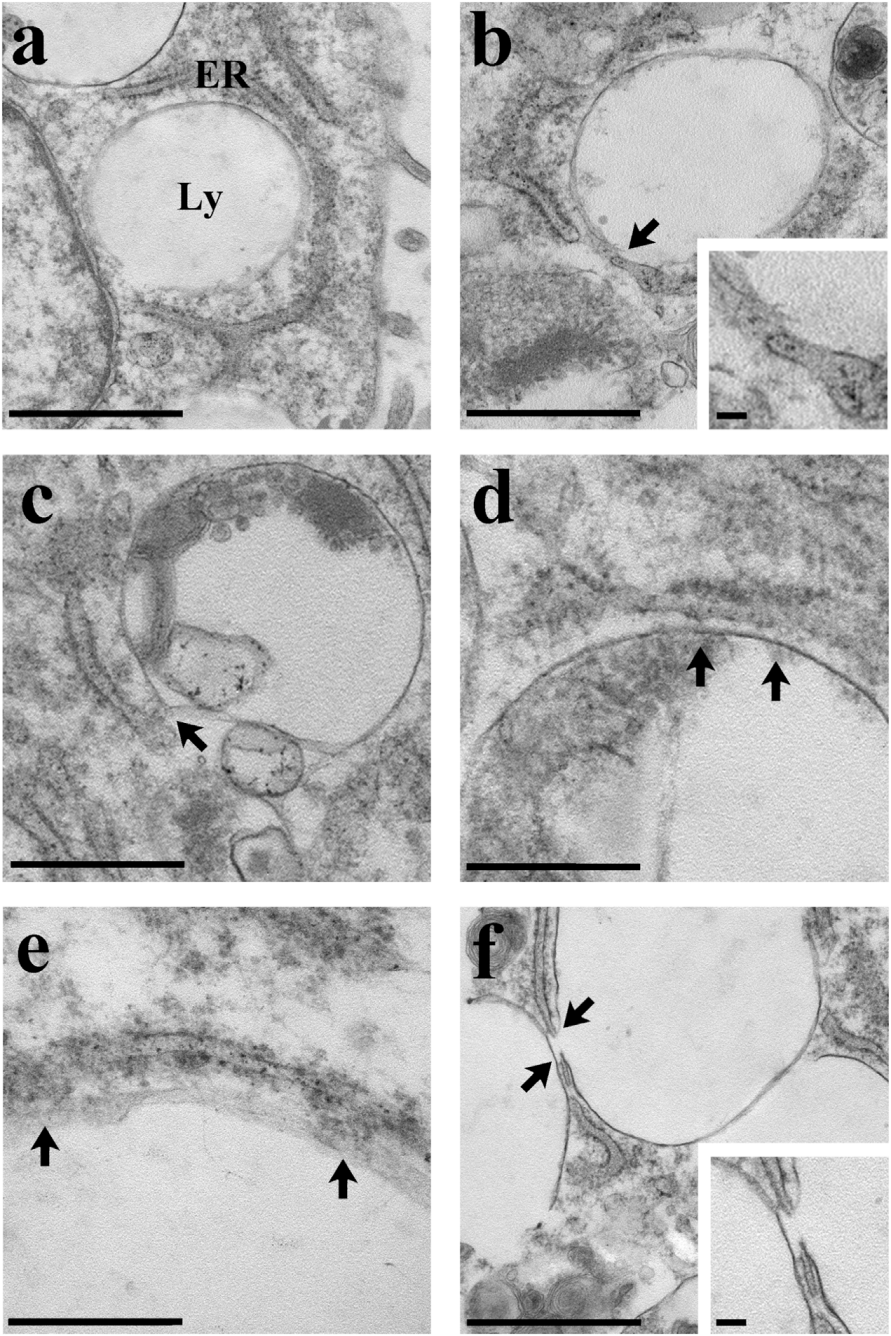
Ultrastructural analysis of C-MSCs. Several examples of membrane contacts between lysosomes (Ly) and ER are shown. **(A)** Note the closeness of the lysosomes with the cell nucleus and ER. Scale bar: 1 µm. **(B)** The lysosome-ER contact site is indicated (arrow and inset). Scale bar: 1 μm; inset scale bar: 100 nm. **(C)** The arrow indicates the membrane contact site. Scale bar: 500 nm. **(D)** Extensive contact (arrows) between the lysosomal and the ER membranes. Scale bar: 500 nm. **(E)** Note the contact (arrows) between the ribosomes-rich ER and the lysosome. Scale bar: 200 nm. **(F)** Two close lysosomes; the one on the right is in tight contact with the ER (arrows and inset). Scale bar: 1 μm; inset scale bar: 100 nm.

The local release of lysosomal Ca^2+^ evoked by NAADP has long been known to be amplified into a global increase in [Ca^2+^]_i_ by the recruitment of juxtaposed InsP_3_Rs on the ER membrane (Churchill and Galione, 2001; Kinnear et al., 2004; Davis et al., 2012; Faris et al., 2019; Moccia et al., 2021b). To assess whether the ER Ca^2+^ store is required to maintain lysosomal Ca^2+^ release, we first exploited cyclopiazonic acid (CPA), an established inhibitor of Sarco-Endoplasmic reticulum Ca^2+^-ATPase activity, as recently shown elsewhere (Kilpatrick et al., 2013; Faris et al., 2019; Moccia et al., 2021b). In the absence of extracellular Ca^2+^ (0Ca^2+^), CPA (30 µM) induced a transient elevation in [Ca^2+^]_i_ due to Ca^2+^ efflux into the cytosol through ER leakage channels followed by Ca^2+^ extrusion across the plasma membrane (Figure 3A). While NAADP-AM (1 µM) was able to induce robust Ca^2+^ release in not-treated cells (Figure 3B), it failed to evoke endogenous Ca^2+^ mobilization upon CPA-induced depletion of the ER Ca^2+^ store (Figure 3A). A preliminary characterization of the Ca^2+^ handling machinery revealed that C-MSCs express InsP_3_Rs, but not RyRs, and that InsP_3_-induced ER Ca^2+^ discharge activates SOCE (Maione et al., 2020a). To assess the contribution of InsP_3_Rs to NAADP-induced intracellular Ca^2+^ mobilization, we adopted a similar strategy to that described in (Kilpatrick et al., 2013; Kilpatrick et al., 2016; Faris et al., 2019; Moccia et al., 2021b). The transient increase in [Ca^2+^]_i_ evoked by NAADP-AM (1 µM) was significantly (*p* < 0.001) reduced by blocking InsP_3_Rs with 2-Aminoethoxydiphenyl borate (2-APB) (50 μM, 30 min) (Figure 3C) (Kilpatrick et al., 2013; Kilpatrick et al., 2016) and was suppressed by inhibiting the basal production of InsP_3_ with U73122 (10 μM, 10 min) (Figure 3C), which selectively interferes with phospholipase C (PLC) activity (Moccia et al., 2006; Negri et al., 2021a). The statistical analysis of these data has been presented in Figure 3D. The lack of full inhibition of NAADP-AM-evoked intracellular Ca^2+^ mobilization could be due to the incomplete inhibition of InsP_3_Rs, as also reported in ECFCs (Moccia et al., 2021b), rat gastric smooth muscle cells (Pereira et al., 2014), and MDA-MB-231 breast cancer cells (Vismara et al., 2021). Therefore, InsP_3_Rs provide a robust source of Ca^2+^ during lysosomal Ca^2+^ mobilization and, based upon previous observations (Davis et al., 2012; Kilpatrick et al., 2013; Ronco et al., 2015; Kilpatrick et al., 2016; Faris et al., 2019; Moccia et al., 2021b), it can be concluded that they can be recruited by CICR upon NAADP-induced lysosomal Ca^2+^ release. TEM was then exploited to assess whether MCSs between lysosomal vesicles and ER cisternae can also be detected and thereby sustain the Ca^2+^-dependent cross-talk between the two organelles also in C-MSCs (Kilpatrick et al., 2013). For this purpose, after glutaraldehyde fixation cells have been post-fixed in aqueous OsO_4_ in order to darkly stain lipids and membranes, as described in Section 2. TEM micrographs indicated extensive ER-lysosome MCSs (<20 nm, 14.3 ± 1.13, *n* = 27 from five cells) with ultrastructural resolution (Figure 4). As also reported in human fibroblasts, in the regions of close appositions (e.g., Figure 4A), fibres that appear to tether lysosomes and ER membranes were clearly discernible. In addition, we detected regions where the apposing membranes appeared to be physically coupled with no visible space between them (e.g., Figures 4B,C). Quantification in random sections showed that 60.5% of lysosomes established contact sites with the ER. As also discussed in Kilpatrick et al. (2013), this is likely to be an underestimate as lysosomal diameter spans between 200 and 500 nm and is, therefore, predicted to extend over several sections above and below the selected plane, where additional contact sites might have been established. Of note, lysosomes could establish extensive contact sites both with the smooth (Figure 4D) and the rough (Figure 4E) ER. We also found that ER cisternae could come in direct contacts with more than one lysosome (Figure 4E). Overall, these findings provide the ultrastructural evidence that the architecture of lysosomes and ER MCSs is fully consistent with the recruitment of ER-embedded InsP_3_Rs by NAADP-induced lysosomal Ca^2+^ release through TPCs.

### 3.4 SOCE Maintains NAADP-AM-Evoked Intracellular Ca^2+^ Signals in Cardiac Mesenchymal Stromal Cells

**FIGURE 5:**
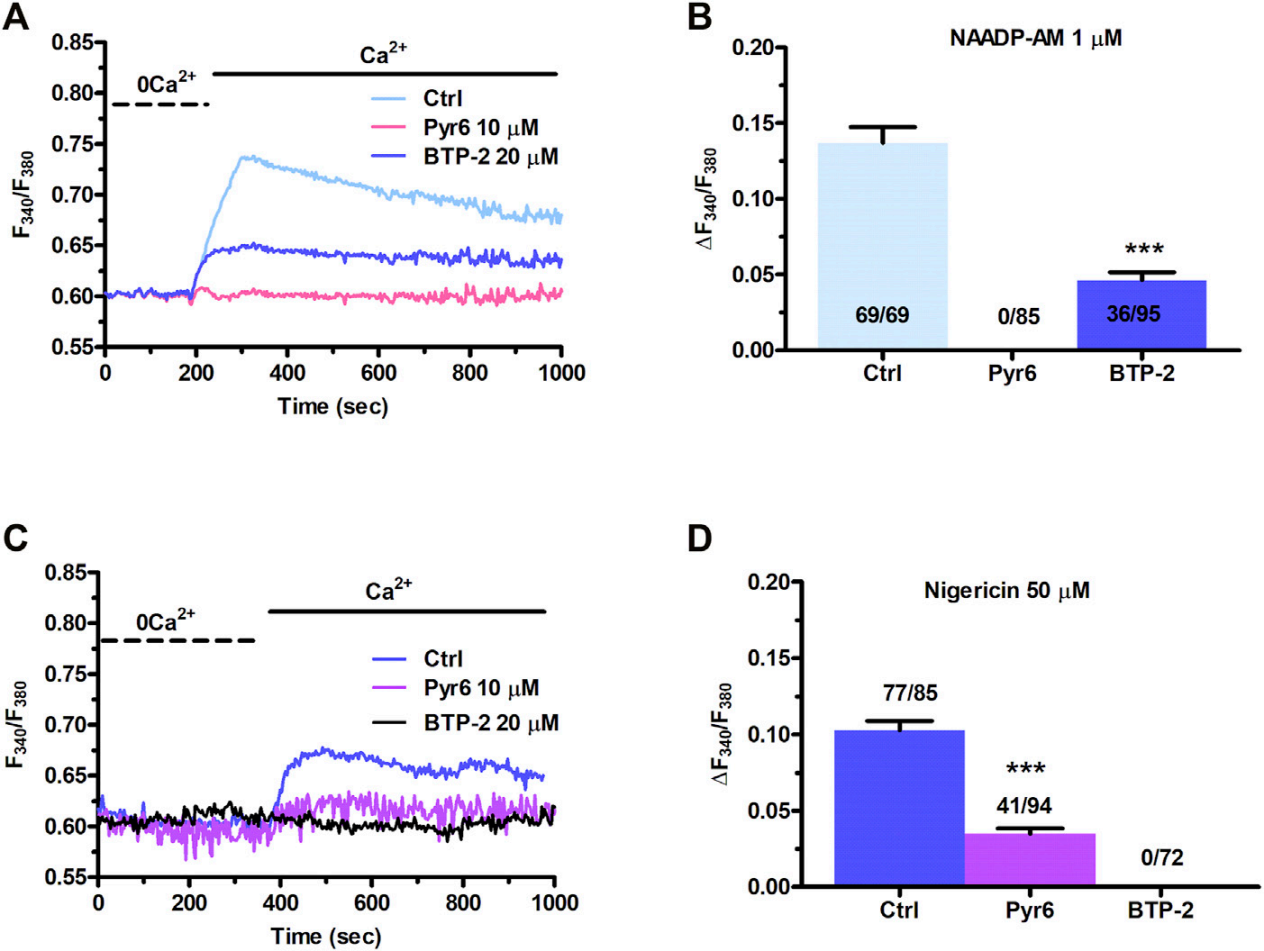
NAADP-AM-induced lysosomal Ca^2+^ mobilization is functionally coupled to SOCE in C-MSCs. **(A)** The influx of extracellular Ca^2+^ evoked by NAADP-AM (1 µM) upon depletion of intracellular Ca^2+^ stores (Ctrl) was severely affected by inhibiting SOCE with Pyr6 (10 μM, 10 min) or BTP-2 (20 μm, 20 min). The previous NAADP-AM-evoked endogenous Ca^2+^ release has not been shown. **(B)** Mean ± SE of the amplitude of NAADP-AM-evoked Ca^2+^ entry evoked by nigericin in the absence (Ctrl) and presence of Pyr-6 and BTP-2. Student’s t-test: ***p < 0.001. **(C)** Nigericin-evoked extracellular Ca^2+^ entry was attenuated or inhibited by, respectively, blocking SOCE with BTP-2 (20 μM, 20 min) or Pyr6 (10 μM, 10 min). **(D)** Mean ± SE of the amplitude of Ca^2+^ entry evoked by nigericin in the absence (Ctrl) and presence of Pyr-6 and BTP-2. Student’s t-test: ***p < 0.001.

Figure 1B clearly shows that NAADP-AM-induced mobilization of intercellularly stored Ca^2+^ resulted in extracellular Ca^2+^ entry even after the agonist washout from the perfusate. This feature clearly hints at SOCE as the Ca^2+^ entry pathway sustaining the long-lasting increase in [Ca^2+^]_i_ evoked by NAADP in the presence of extracellular Ca^2+^. Indeed, InsP_3_-dependent ER Ca^2+^ mobilization results in SOCE activation virtually in all mammalian cells (Prakriya and Lewis, 2015; Emrich et al., 2021), including C-MSCs (Maione et al., 2020a). In order to assess whether NAADP-AM-induced lysosomal Ca^2+^ release can lead to SOCE *via* intermediate ER Ca^2+^ depletion, we repeated the “Ca^2+^ add-back” protocol described in Figure 1 in the absence and presence of BTP-2 or Pyr6, two selective blockers of SOCE (Schleifer et al., 2012; Moccia et al., 2016). This strategy has long been exploited to selectively evaluate the blocking effect of SOCE-targeting drugs on agonist-evoked extracellular Ca^2+^ entry rather than on the previous phase of endogenous Ca^2+^ mobilization (Sanchez-Hernandez et al., 2010; Jairaman et al., 2015; Rahman and Rahman, 2017; Scarpellino et al., 2019; Negri et al., 2020; Schach et al., 2020). The influx of Ca^2+^ secondary to Ca^2+^ restitution to the perfusate after removal of NAADP-AM (Figure 5A) from the perfusate was significantly (*p* < 0.001) attenuated by BTP-2 (20 μM, 20 min) and abrogated by Pyr6 (10 μM, 10 min) (Figure 5A). The statistical analysis of these data has been presented in Figure 5B. These observations demonstrate that NAADP-induced lysosomal Ca^2+^ mobilization in C-MSCs is functionally coupled to SOCE *via* InsP_3_-dependent ER Ca^2+^ release. Therefore, lysosomal Ca^2+^ release must induce depletion of ER Ca^2+^ *via* InsP_3_Rs, thereby leading to SOCE recruitment on the plasma membrane. To further support this conclusion, Supplementary Figure S5A shows that also the pharmacological depletion of the lysosomal Ca^2+^ store with nigericin (50 µM) induced both intracellular Ca^2+^ release and extracellular Ca^2+^ entry. Furthermore, the intracellular Ca^2+^ response to nigericin (50 µM) was significantly (*p* < 0.001) reduced by blocking InsP_3_Rs with 2-APB (50 μM, 30 min) (Supplementary Figures S5B,C) and by interfering with basal InsP_3_ production with U73122 (10 μM, 10 min) (Supplementary Figures S5B,C), as recently shown in primary cultures of colorectal cancer cells (Faris et al., 2019) and in circulating ECFCs (Moccia et al., 2021b). Finally, nigericin-evoked extracellular Ca^2+^ entry was significantly (*p* < 0.001) attenuated by blocking SOCE with either BTP-2 (20 μM, 20 min) or Pyr6 (10 μM, 10 min) (Figures 5C,D). This result is, therefore, consistent with the evidence reported above that NAADP-induced Ca^2+^ release through TPCs is able to induce ER Ca^2+^ depletion followed by SOCE activation.

### 3.5 Nicotinic Acid Adenine Dinucleotide Phosphate-Induced Lysosomal Ca^2+^ Release *via* TPCs Supports FBS-Induced Intracellular Ca^2+^ Oscillations in C-MSCs

**FIGURE 6:**
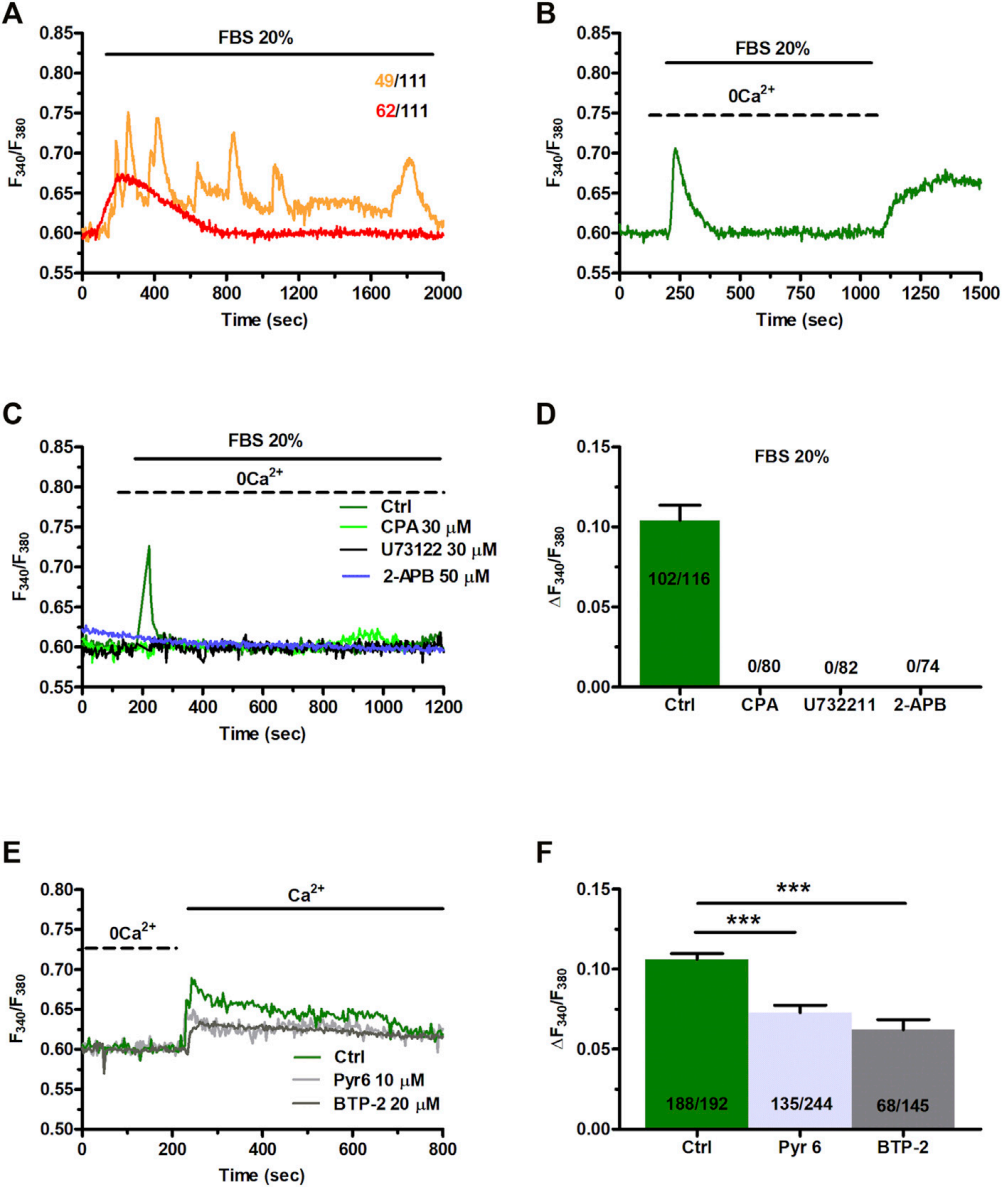
Fetal bovine serum (FBS)-induces intracellular Ca^2+^ release from endogenous stores and enhance SOCE. **(A)** 20% FBS induced oscillatory or transient increases in [Ca^2+^]_i_. **(B)** Under 0Ca^2+^ conditions, 20% FBS induced a transient elevation in [Ca^2+^]_i_. Subsequent re-addition of extracellular Ca^2+^, 100 s after agonist removal from the bath, resulted in a second bump in [Ca^2+^]_i_ that was indicative of SOCE. **(C)** The intracellular Ca^2+^ release evoked by 20% FBS (Ctrl) was inhibited by depleting the ER Ca^2+^ pool with CPA (30 μM, 30 min), by blocking InsP_3_Rs with 2-APB (50 μM, 30 min), or inhibiting PLC with U73122 (10 μM, 10 min). **(D)** Mean ± SE of the amplitude of the peak intracellular Ca^2+^ response to 20% FBS under the designated treatments. **(E)** Subsequent to store depletion by 20% of FBS application (data are not shown her**(E)**, FBS were washed out from bath, then extracellular Ca^2+^ added to the bath to bath in the presence and absence of SOCE inhibitors, Pyr6 (10 μm, 10 min) or BTP-2 (20 μm, 20 min). **(F)** Mean ± SE of the amplitude of Ca^2+^ entry evoked by 20% FBS in the absence (Ctrl) and presence of Pyr6 and BTP-2. The asterisk indicates ***p < 0.001.

FBS has been shown to induce intracellular Ca^2+^ signals to stimulate proliferation in primary MSCs harvested from rat bone marrow (Foreman et al., 2006). 20% FBS induced intracellular Ca^2+^ oscillations also in ≈26% of C-MSCs, whereas it promoted a transient increase elevation in [Ca^2+^]_i_ in the remaining 74% cells (Figure 6A). Intracellular Ca^2+^ oscillations lasted for at least 30 min, while the transient Ca^2+^ signal took approximately 13 min to decline to pre-stimulation levels (Figure 6A). In the absence of extracellular Ca^2+^ (0Ca^2+^), 20% FBS induced a rapid (≈3 min) increase in [Ca^2+^]_i_ that reflected endogenous Ca^2+^ mobilization. The subsequent re-addition of extracellular Ca^2+^, 100 s after FBS removal from the bath, resulted in a second bump in [Ca^2+^]_i_, which was due to extracellular Ca^2+^ entry and was likely to be mediated by SOCE (Figure 6B). FBS-induced intracellular Ca^2+^ signals are known to be triggered by InsP_3_-induced ER Ca^2+^ mobilization and maintained over time by SOCE (Foreman et al., 2006; Hu et al., 2009; Zuccolo et al., 2018b). Preliminary experiments confirmed that 20% FBS-induced intracellular Ca^2+^ release was abrogated by depleting the ER Ca^2+^ store with CPA (30 μM, 30 min) (Figures 6C,D), inhibiting InsP_3_Rs with 2-APB (50 μM, 30 min) (Figures 6C,D), and blocking PLC with U73122 (10 μM, 10 min) (Figures 6C,D). Furthermore, 20% FBS-induced extracellular Ca^2+^ entry was significantly (*p* < 0.001) reduced by inhibiting SOCE with BTP-2 (20 μM, 20 min) or Pyr6 (10 μM, 10 min) (Figures 6E,F).

**FIGURE 7:**
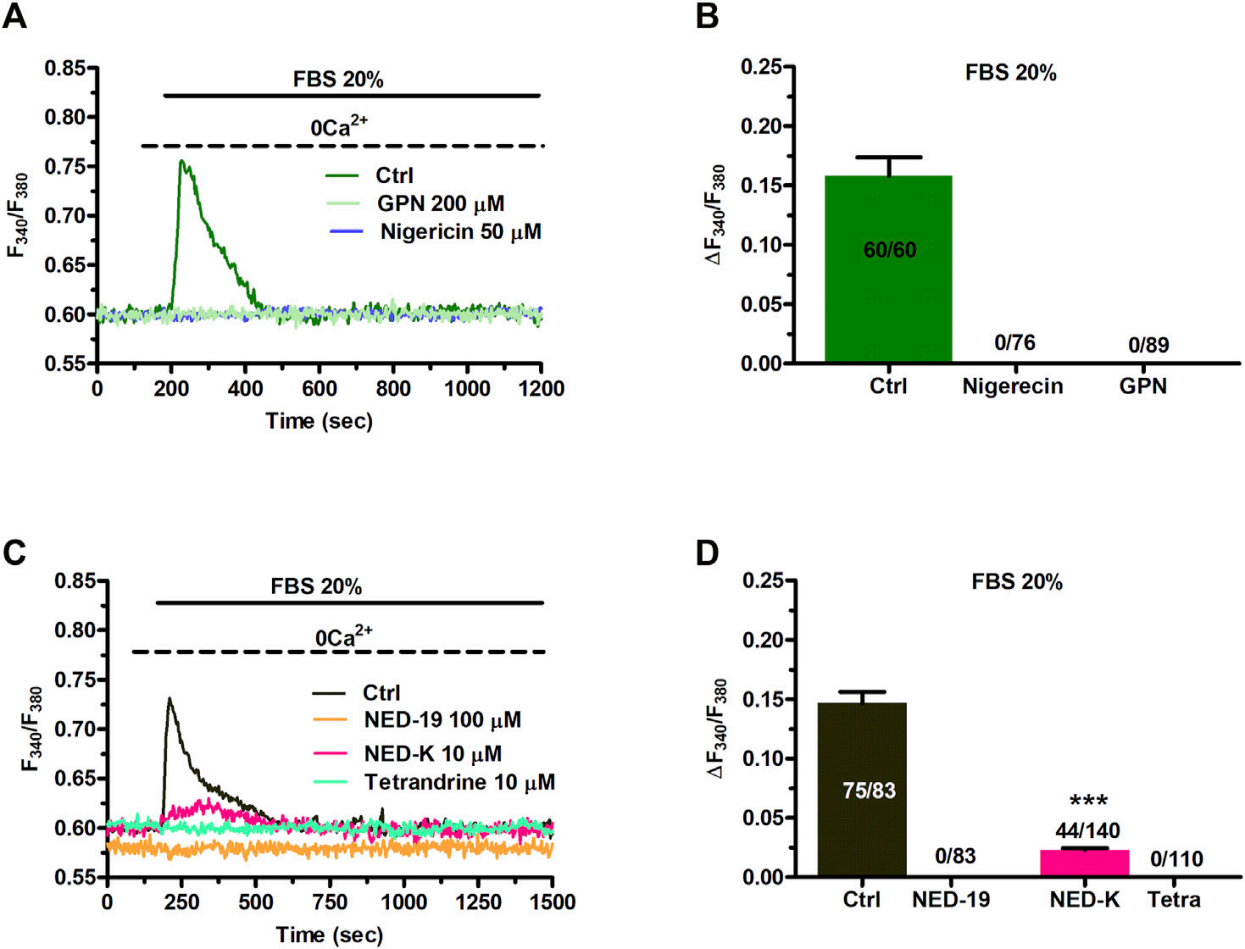
TPCs mediate 20% FBS-induced intracellular Ca^2+^ release. **(A)** Intracellular Ca^2+^ release induced by 20% FBS was abolished upon depletion of the lysosomal Ca^2+^ pool with either GPN (200 μM, 30 min) or nigericin (50 μM, 30 min). **(B)** Mean ± SE of the amplitude of the intracellular Ca^2+^ peak evoked by 20% FBS under the designated treatments. **(C)** 20% FBS induced an intracellular Ca^2+^ transient that was significantly reduced or inhibited by blocking TPCs with, respectively, NED-K (10 μM, 30 min), tetrandrine (10 μM, 30 min) or NED-19 (100 μM, 30 min). **(D)** Mean ± SE of the amplitude of the intracellular Ca^2+^ peak evoked by 20% FBS in the absence (Ctrl) or presence of NED-19, NED-K or tetrandrine (Tetra). Student’s t-test: ***p < 0.001.

The evidence reported above clearly showed that NAADP-induced lysosomal Ca^2+^ release via TPCs was able to promote InsP_3_-induced Ca^2+^ release from the ER, thereby resulting in SOCE activation on the plasma membrane. Therefore, we sought to assess the role of NAADP-induced lysosomal Ca^2+^ release in the Ca^2+^ response to 20% FBS (Figure 7A). The depletion of the lysosomal Ca^2+^ store with either GPN (200 μM, 30 min) (Figures 7A,B) or nigericin (50 μM, 30 min) (Figures 7A,B) abrogated FBS-induced intracellular Ca^2+^ mobilization. The same effect was achieved upon pharmacological blockade of TPCs with NED-19 (100 μM, 30 min) (Figures 7C,D), NED-K (10 μM, 30 min) (Figures 7C,D), and tetrandrine (Figures 7C,D). Therefore, NAADP plays a crucial role in igniting the Ca^2+^ response to 20% FBS in C-MSCs.

### 3.6 TPCs Mediate 20% FBS-Induced Proliferation and ERK Phosphorylation in Cardiac Mesenchymal Stromal Cells

**FIGURE 8:**
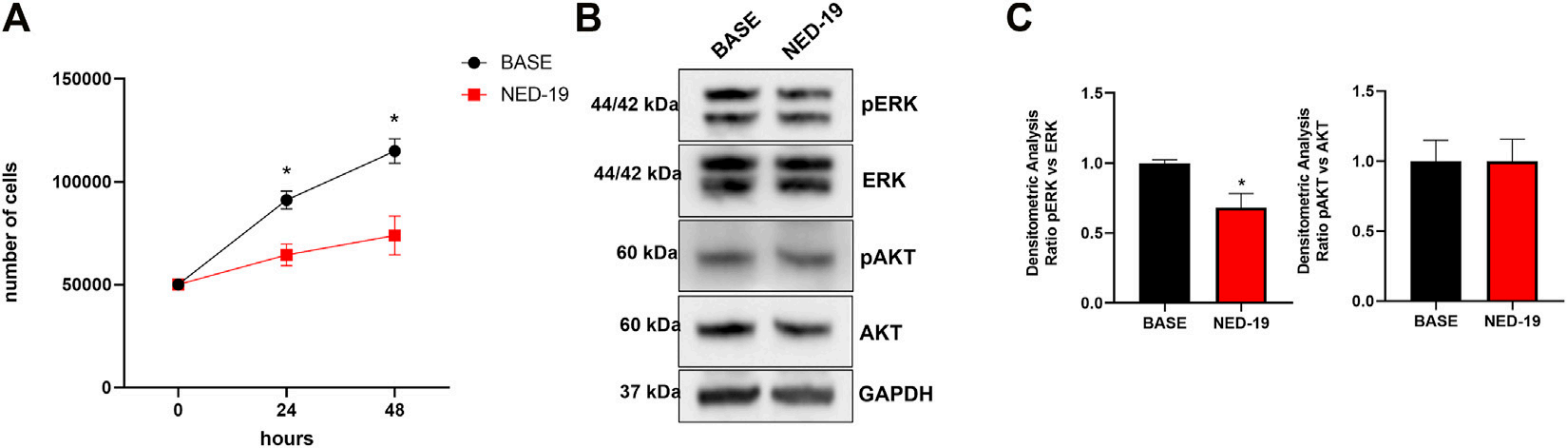
TPCs mediate 20% FBS-induced proliferation and ERK phosphorylation in C-MSCs. Following 4 h of growth without serum, cells were treated with NED-19 (100 μM, 30 min) and subsequently stimulated with 20% FBS. **(A)** Following 24 and 48 h of FBS stimulation, the medium was removed, cells detached from the plates, and counted by hemocytometer (*n* = 3/group). Student’s t-test: *p < 0.05. **(B)** The cells were lysate after 60 min of FBS stimulation. Total protein extract from treated cells was subjected to Western blot analysis to visualize active phosphorylated form and total of ERK and AKT using specific antibodies. Phospho-ERK1/2 and Phospho-AKT levels were corrected by total ERK1/2 and AKT densitometry respectively. **(C)** Western blot data are presented as the fold change of target protein expression. The results are expressed as mean ± SEM (*n* = 3/group). Student’s t-test: *p < 0.05.

In order to assess the physiological role of NAADP-induced lysosomal Ca^2+^ release through TPCs, 20% FBS-induced C-MSC proliferation was evaluated in the absence (Ctrl) and presence of NED-19 (100 μM, 30 min). Figure 8A shows that the pharmacological blockade of TPCs significantly (*p* < 0.05) reduced the total cell number at 24 and 48 h, thereby showing the crucial role of TPCs in supporting C-MSC proliferation. Flow cytometric analysis of Annexin V fluorescence confirmed that pre-treating C-MSCs with NED-19 did not induce apoptosis (Supplementary Figure S6). In order to determine whether TPCs recruit mitogen-associated protein kinases (MAPKs), we evaluated the phosphorylated levels of the Ca^2+^-dependent extracellular signal-regulated protein kinases 1 and 2 (ERK1/2) and of the survival kinase, Akt (Zuccolo et al., 2018a; Faris et al., 2019; Negri et al., 2021b). Figures 8B,C illustrate that 20% FBS-induced ERK1/2, but not Akt, phosphorylation was significantly (*p* < 0.05) inhibited by blocking TPCs with NED-19 (100 μM, 30 min). Overall, these findings demonstrate that NAADP-induced lysosomal Ca^2+^ release through TPCs stimulates C-MSC proliferation by engaging ERK1/2.

## 4 Discussion

NAADP is emerging as a crucial regulator of intracellular Ca^2+^ signalling and Ca^2+^-dependent processes in the cardiovascular system (Macgregor et al., 2007; Collins et al., 2011; Fameli et al., 2017; Moccia et al., 2021a). C-MSCs represent the large majority of supportive cells in the heart, are critical to normal cardiac function and contribute to maladaptive cardiac remodelling under multiple pathological conditions. Herein, we showed for the first time that NAADP mobilizes EL Ca^2+^ *via* TPCs also in C-MSCs. NAADP-evoked intracellular Ca^2+^ signals are amplified by InsP_3_-sensitive ER Ca^2+^ release at lysosomes-ER C-MCSs followed by SOCE activation. The functional crosstalk between NAADP-evoked lysosomal Ca^2+^ release, InsP_3_-induced ER Ca^2+^ mobilization and SOCE sustains FBS-induced intracellular Ca^2+^ signals and proliferation by promoting ERK phosphorylation.

### 4.1 Nicotinic Acid Adenine Dinucleotide Phosphate Evokes Complex Ca^2+^ Signals in Cardiac Mesenchymal Stromal Cells

Intracellular Ca^2+^ signals tightly control a plethora of crucial functions in human MSCs from multiple sources, as reviewed in Moccia et al. (2015), Forostyak et al. (2016), and Jiang et al. (2017). According to the canonical model, the Ca^2+^ response evoked by chemical stimulation in human MSCs is triggered by InsP_3_-dependent ER Ca^2+^ and may be maintained over time by SOCE (Kawano et al., 2002; Peng et al., 2016; Kotova et al., 2018). Herein, we expanded our knowledge of the molecular mechanisms shaping intracellular Ca^2+^ signalling in these cells by focusing on the role played by NAADP in C-MSCs. The intracellular delivery of NAADP mobilizes acidic Ca^2+^ stores throughout the cardiovascular system (Moccia et al., 2021a), e.g., in guinea pig ventricular (Macgregor et al., 2007) and atrial (Collins et al., 2011) cardiomyocytes, rat pulmonary artery VSMCs (Kinnear et al., 2004), human aortic endothelial cells (Brailoiu et al., 2010), mouse brain endothelial cells (Zuccolo et al., 2019), and circulating ECFCs (Di Nezza et al., 2017; Moccia et al., 2021b). Likewise, NAADP-AM, a membrane-permeable analogue of NAADP, could induce either a transient elevation in [Ca^2+^]_i_ or a burst of intracellular Ca^2+^ oscillations. This latter observation is in accord with the evidence that: 1) intracellular delivery of NAADP may induce oscillatory Ca^2+^ signals in human Jurkat T-lymphocytes (Berg et al., 2000), cytotoxic T lymphocytes (Davis et al., 2012), and human pancreatic β-cells (Johnson and Misler, 2002); 2) NAADP contributes to agonist-induced repetitive Ca^2+^ spikes in several types of endothelial cells (Zuccolo et al., 2019; Berra-Romani et al., 2020; Balducci et al., 2021), and that 3) NAADP induces intracellular Ca^2+^ oscillations in mouse cardiomyocytes during reperfusion injury (Davidson et al., 2015). Early work conducted on echinoderms first suggested that NAADP was able to elicit repetitive Ca^2+^ oscillations by promoting a Ca^2+^-dependent crosstalk between two different Ca^2+^ pools (Churchill and Galione, 2001), which were later shown to be located in acidic vesicles and ER (Churchill et al., 2002; Moccia et al., 2006).

### 4.2 Nicotinic Acid Adenine Dinucleotide Phosphate-Induced Intracellular Ca^2+^ Signals Are Triggered by Lysosomal Ca^2+^ Release *via* TPCs, Amplified by InsP_3_-Evoked ER Ca^2+^ Release and Maintained by SOCE

The Ca^2+^ response to NAADP in C-MSCs comprised an early phase of intracellular Ca^2+^ mobilization followed by a later phase of extracellular Ca^2+^ entry, which required the previous depletion of the endogenous Ca^2+^ pool but not the NAADP-AM presence in the perfusate. First, we found that GPN, nigericin, and bafilomycin A1, which provide three established pharmacological tools to mobilize acidic Ca^2+^ stores (Kilpatrick et al., 2013; Ronco et al., 2015; Morgan and Galione, 2021; Yuan et al., 2021), prevent NAADP-induced intracellular Ca^2+^ mobilization. In agreement with the hypothesis that the lysosomal compartment represents the primary source of this increase in [Ca^2+^]_i_, all of these drugs, as well as NH_4_Cl, induced a rapid reduction in Lysotracker Red fluorescence. Although a recent investigation questioned the documented GPN ability to release lysosomal Ca^2+^ (Atakpa et al., 2019), Patel’s group provided the clear-cut evidence that this compound mobilizes Ca^2+^ from acidic organelles and may, therefore, be safely exploited to probe the endogenous store primarily targeted by NAADP (Morgan et al., 2020; Yuan et al., 2021). We further showed that C-MSCs express both TPC1 and TPC2 transcripts and proteins, and that the Ca^2+^ response to NAADP was inhibited by blocking TPCs with two selective antagonists, such as NED-19 (Macgregor et al., 2007; Di Nezza et al., 2017; Jin et al., 2020; Moccia et al., 2021a) and NED-K (Davidson et al., 2015), and the traditional Chinese herbal remedy, tetrandrine, which can target both TPC1 and TPC2 (Sakurai et al., 2015; Moccia et al., 2021a). As recently reviewed in Moccia et al. (2021a) and Negri et al. (2021b), TPC1 and TPC2 are both present in mouse ventricular cardiomyocytes, but this is the first time that they were reported in any other cellular component of the human heart. As reviewed in Pitt et al. (2016), TPC1 presents a limited Ca^2+^ permeability, while TPC2 is predicted to release more Ca^2+^ upon activation. Nevertheless, it has been shown that even a small Ca^2+^ flux through TPC1 can generate a global increase in [Ca^2+^]_i_ when lysosomal vesicles are juxtaposed to ER cisternae (Galione, 2019). For instance, TPC1 alone supports NAADP-induced intracellular Ca^2+^ oscillations in circulating ECFCs (Di Nezza et al., 2017; Moccia et al., 2021b) and in mouse cardiac myocytes undergoing the ischemia-reperfusion injury (Davidson et al., 2015). Furthermore, TPC1 was sufficient to maintain the intracellular Ca^2+^ response to nutrients or incretins in mouse pancreatic β cells deficient for TPC2 (Cane et al., 2016). Three pieces of evidence suggest that InsP_3_Rs in ER cisternae contribute to amplify NAADP-induced lysosomal Ca^2+^ release. First, depletion of the ER Ca^2+^ pool with CPA suppressed or attenuated the intracellular Ca^2+^ release evoked by both NAADP and the H^+^/K^+^ antiporter, nigericin. Second, NAADP-induced endogenous Ca^2+^ mobilization was impaired by inhibiting InsP_3_Rs with 2-APB and by blocking basal InsP_3_ production with U73122. Conversely, functional RyRs are absent in C-MSCs (Maione et al., 2020a). The requirement for InsP_3_Rs to sustain the increase in [Ca^2+^]_i_ resulting from NAADP-AM-evoked Ca^2+^ release from lysosomal vesicles is in full agreement with previous work carried out on human fibroblasts (Kilpatrick et al., 2013), human ECFCs (Moccia et al., 2021b), COS-7 cells (Morgan and Galione, 2021), HeLa cells (Ronco et al., 2015), and human metastatic colorectal cancer cells (Faris et al., 2019). Third, TEM revealed clearly discernible ER-lysosomes MCSs, which closely resemble those previously described in human fibroblasts (Kilpatrick et al., 2013) and could provide the sub-cellular framework to enable InsP_3_R recruitment by local Ca^2+^ release through TPCs (Penny et al., 2014). Likewise, the MCSs between lysosomal vesicles and ER cisternae in C-MSCs are similar to the cytoplasmic nanojunctions between lysosomes and sarcoplasmic reticulum (SR) recently reported in rat aortic VSMCs (Fameli et al., 2014).

The different extent of coupling between lysosomal TPCs and ER-embedded InsP_3_Rs (due to changes in either their distribution or density at MCSs) could explain the onset of a long-lasting elevation in [Ca^2+^]_i_ that replaces the intracellular Ca^2+^ oscillations in a fraction of C-MSCs. For instance, computational modelling indicated that TPC clustering within the microdomain could accelerate the frequency of InsP_3_Rs-driven Ca^2+^ oscillations (Penny et al., 2014), which could ultimately lead to the fusion of the Ca^2+^ spikes and the occurrence of a single, broader increase in [Ca^2+^]_i_ (Bartlett et al., 2020).

Removal of extracellular Ca^2+^ shortened the duration of the Ca^2+^ response to NAADP-AM. Therefore, NAADP is predicted to gate a Ca^2+^-permeable pathway on the plasma membrane. This observation is supported by the evidence that restitution of extracellular Ca^2+^ following exposure to NAADP (or nigericin) under 0Ca^2+^ conditions, results in a second bump in [Ca^2+^]_i_ that reflects extracellular Ca^2+^ entry. This influx of Ca^2+^ occurs after washout of the agonist from the bath and, therefore, it is exclusively coupled to the previous depletion of endogenous Ca^2+^ stores. As discussed elsewhere (Yamazaki et al., 2007; Sanchez-Hernandez et al., 2010; Negri et al., 2020), this feature hints at SOCE as being responsible for NAADP-induced extracellular Ca^2+^ entry. In agreement with this hypothesis, NAADP-evoked Ca^2+^ influx was remarkably reduced in the presence of either BTP-2 or Pyr6, two different inhibitors of Orai1 channels, which provide the pore-forming subunit of store-operated Ca^2+^ channels in non-excitable cells (Prakriya and Lewis, 2015; Emrich et al., 2021) and MSCs (Lee et al., 2016; Peng et al., 2016). SOCE activation ultimately results from the reduction of ER Ca^2+^ concentration (Emrich et al., 2021). As discussed elsewhere (Davis et al., 2012; Brailoiu and Brailoiu, 2016), the engagement of SOCE by NAADP (and nigericin) hints at the depletion of the ER Ca^2+^ content as the intermediate step between lysosomal Ca^2+^ release and extracellular Ca^2+^ entry. However, extracellular Ca^2+^ entry directly evoked by NAADP delivery was not always engaged during acidic Ca^2+^ signalling in the cell types where this functional interplay has been investigated (Faris et al., 2019; Moccia et al., 2021b). Therefore, it is conceivable that lysosomal Ca^2+^ release recruits ER sub-domains that are functionally coupled to the SOCE machinery in C-MSCs, but not in other cell types, as widely discussed in Parekh and Putney (2005). These observations hint at NAADP as a Ca^2+^-releasing second messenger that can trigger a functional crosstalk among multiple Ca^2+^ sources (lysosomes, ER, and plasma membrane) in C-MSCs. In these cells, NAADP may serve as a provider of the “trigger” Ca^2+^ response to extracellular stimulation that is subsequently amplified by InsP_3_Rs on the ER and maintained over time by SOCE activation on the plasma membrane, as previously reported in many mammalian cells, including human fibroblasts (Kilpatrick et al., 2013), human ECFCs (Moccia et al., 2021b) and brain microvascular endothelial cells (Zuccolo et al., 2019), human metastatic colorectal cancer cells (Faris et al., 2019), human primary CTL cells (Davis et al., 2012), and rat pulmonary artery VSMCs (Kinnear et al., 2004).

### 4.3 Lysosomal Ca^2+^ Release *via* TPCs is Crucial to FBS-Induced Intracellular Ca^2+^ Signalling and Proliferation in Cardiac Mesenchymal Stromal Cells

It has long been known that FBS stimulates proliferation through an increase in [Ca^2+^]_i_ that can adopt either a biphasic (Faris et al., 2019) or an oscillatory pattern (Tao et al., 2011) in a variety of cell types, including rat bone marrow MSCs (Foreman et al., 2006). FBS-induced intracellular Ca^2+^ signals are known to impinge on the interplay between InsP_3_-induced Ca^2+^ release from the ER and SOCE (Foreman et al., 2006; Hu et al., 2009). Intriguingly, a recent investigation reported the first evidence that NAADP-evoked lysosomal Ca^2+^ release via TPC1 interacts with InsP_3_-dependent ER Ca^2+^ mobilization and SOCE to promote FBS-induced proliferation in human metastatic colorectal cancer cells (Faris et al., 2019). Unveiling the molecular mechanisms that drive C-MSC proliferation is crucial to improve the therapeutic outcome of regenerative strategies aiming at utilizing these cells to promote cardiac repair (Bagno et al., 2018; Braunwald, 2018). Preliminary analysis showed that FBS evoked a complex increase in [Ca^2+^]_i_ also in C-MSCs, which displayed either an oscillatory or a biphasic Ca^2+^ signal. Pharmacological manipulation confirmed that the Ca^2+^ response to FBS comprised InsP_3_-induced ER Ca^2+^ mobilization followed by SOCE activation. Indeed, FBS-induced intracellular Ca^2+^ release was suppressed by inhibiting InsP_3_Rs with 2-APB, by blocking basal InsP_3_ production with U73122 and by depleting the ER Ca^2+^ store with CPA, whereas FBS-induced extracellular Ca^2+^ entry was remarkably attenuated by blocking SOCE with BTP-2 and Pyr6. Next, we provided the evidence that the NAADP-sensitive acidic Ca^2+^ store is crucial to FBS-induced intracellular Ca^2+^ signals and proliferation in C-MSCs. Indeed, FBS-induced intracellular Ca^2+^ release was abrogated by depleting the lysosomal Ca^2+^ store with either GPN or nigericin, as previously shown in human metastatic colorectal cancer cells (Faris et al., 2019). In agreement with these observations, the selective blockade of TPCs with NED-19, NED-K or tetrandrine also abolished the intracellular Ca^2+^ response to FBS. Therefore, NAADP-induced lysosomal Ca^2+^ release is indispensable to trigger the cytosolic Ca^2+^ response to FBS and this requires the functional recruitment of InsP_3_Rs on the ER *via* CICR at lysosomal-ER MCSs. That the ER is depleted *via* InsP_3_Rs-mediated ER Ca^2+^ release following NAADP-induced lysosomal Ca^2+^ mobilization in response to FBS is also suggested by FBS-induced SOCE activation, which requires a reduction in ER Ca^2+^ concentration (Brailoiu et al., 2009; Davis et al., 2012). The mechanism whereby FBS stimulation results to intracellular NAADP generation in C-MSCs, as well as in human metastatic cancer cells (Faris et al., 2019), remains to be elucidated. Nevertheless, FBS is likely to engage the multifunctional enzyme CD38, which catalyses the “base exchange” of the nicotinamide moiety of NADP with nicotinic acid, thereby resulting in NAADP production in most cell types (Galione, 2015), including cardiomyocytes (Negri et al., 2021b). A recent paper suggested that the dual NADPH oxidases, DUOX1 and DUOX2, contribute to NAADP biosynthesis in murine T lymphocytes (Gu et al., 2021), but their role in NAADP-dependent Ca^2+^ response to FBS is yet to be investigated.

The physiological role of NAADP-induced intracellular Ca^2+^ signals were further assessed by evaluating the effect of NED-19 on C-MSC proliferation. The pharmacological blockade of TPCs with NED-19 strongly reduced FBS-induced C-MSC proliferation at 24 and 48 h. Preliminary experiments indicated that the massive release of Ca^2+^ induced by nigericin *per se* resulted in C-MSC cell death already at 24 h from exposure to this lysosomotropic compound. While this observation is in accord with the reported effects of nigericin on various cell models (Murakami et al., 2012), it prevented us from probing its ability to interfere with FBS-induced proliferation. Previous work showed that NAADP-induced Ca^2+^ release may stimulate proliferation by recruiting the Ca^2+^-dependent ERK1/2 and Akt signalling pathways (Faris et al., 2019; Negri et al., 2021b). Consistently, FBS-induced ERK1/2 phosphorylation was impaired by the pharmacological blockade of TPCs with NED-19, whereas Akt engagement was unaffected. Interestingly, ERK1/2, but not Akt, was harnessed by intracellular Ca^2+^ oscillations to drive FBS-induced proliferation also in human bone marrow MSCs (Tao et al., 2011). Additionally, NAADP-induced intracellular Ca^2+^ oscillations could underpin another crucial function of C-MSCs, i.e., the regulation of extracellular matrix (ECM) composition (Maione et al., 2020b). For instance, bone marrow-derived human MSCs exhibited repetitive Ca^2+^ spikes during aligned collagen matrix formation (Gilchrist et al., 2019), whereas extracellular Ca^2+^ entry in human airway epithelial cells drives the expression and secretion of matrix-degrading enzymes, such as matrix metalloprotease 1 (Li et al., 2011). Interestingly, an increase in [Ca^2+^]_i_ in cardiac fibroblasts may also regulate collagen remodelling in mouse hearts (Adapala et al., 2020). Therefore, future studies will have to assess the role of NAADP-induced Ca^2+^ signalling in the modulation of ECM composition by C-MSCs.

In conclusion, this study demonstrated that NAADP induces intracellular Ca^2+^ signals in C-MSCs by promoting lysosomal Ca^2+^ release *via* TPCs that is in turn amplified by ER-embedded InsP_3_Rs at lysosomal-ER MCSs. The following depletion of the ER Ca^2+^ pool activates SOCE, which prolongs the Ca^2+^ response to NAADP. FBS impinges on the NAADP-induced Ca^2+^-dependent crosstalk between lysosomes and ER to stimulate proliferation through the Ca^2+^-dependent ERK1/2 signalling pathway. These findings pave the way for future studies assessing whether NAADP signalling in C-MSCs could be targeted to favour cardiac repair upon an ischemic insult or to other pathologies associated to maladaptive cardiac remodelling, such as ACM, heart failure and cardiac fibrosis.

## Data Availability

The raw data supporting the conclusion of this article will be made available by the authors, without undue reservation.
