# Supplementary material for: Nicotinic Acid Adenine Dinucleotide Phosphate Induces Intracellular Ca2+ Signalling and Stimulates Proliferation in Human Cardiac Mesenchymal Stromal Cells
Source: Front Cell Dev Biol. 2022 Mar 15;10:874043. doi: 10.3389/fcell.2022.874043 (PMC8980055; doi:10.3389/fcell.2022.874043)
Supplement: Supplementary file 1 [file DataSheet1.docx]

Supplementary Material

# Supplementary Materials and methods

**1.1 [Ca^2+^]_i_ measurements**

We utilized the Ca^2+^ imaging set-up that we have described elsewhere [[1](#_ENREF_1)]. C-MSCs were loaded with 2 µM Fura-2 acetoxymethyl ester (Fura-2/AM; 1 mM stock in dimethyl sulfoxide) in PSS for 30 min at 37°C and 5% CO_2_ saturated humidity. After washing in PSS, the coverslip was fixed to the bottom of a Petri dish and the cells observed by an upright epifluorescence Axiolab microscope (Carl Zeiss, Oberkochen, Germany), usually equipped with a Zeiss ×40 Achroplan objective (water-immersion, 2.0 mm working distance, 0.9 numerical aperture). The cells were excited alternately at 340 and 380 nm, and the emitted light was detected at 510 nm. A first neutral density filter (1 or 0.3 optical density) reduced the overall intensity of the excitation light, and a second neutral density filter (optical density=0.3) was coupled to the 380 nm filter to approach the intensity of the 340 nm light. A round diaphragm was used to increase the contrast. The excitation filters were mounted on a filter wheel (Lambda 10, Sutter Instrument, Novato, CA, USA). Custom software, working in the LINUX environment, was used to drive the camera (Extended-ISIS Camera, Photonic Science, Millham, UK) and the filter wheel, and to measure and plot on-line the fluorescence from 15-25 rectangular “regions of interest” (ROI) enclosing a corresponding number of single cells. Each ROI was identified by a number. Adjacent ROIs never superimposed. [Ca^2+^]_i_ was monitored by measuring, for each ROI, the ratio of the mean fluorescence emitted at 510 nm when exciting alternatively at 340 and 380 nm [Ratio (F_340_/F_380_)]. An increase in [Ca^2+^]_i_ causes an increase in the ratio [[2](#_ENREF_2)]. Ratio measurements were performed and plotted on-line every 3 s. The experiments were performed at room temperature (22°C).

**1.2 Lysotracker red measurements**

The lysosomal compartments were visualized in vital cells by incubating the C-MSCs with LysoTraker Red, as described in [[3](#_ENREF_3), [4](#_ENREF_4)]. Briefly, cells were incubated with Lysotracker Red (100 nM) for 20 min at 37 ◦C. To visualize lysosomes, the cells observed by an upright epifluorescence Axiolab microscope (Carl Zeiss, Oberkochen, Germany), usually equipped with a Zeiss × 40 Achroplan objective (water-immersion, 2.0 mm working distance, 0.9 numerical aperture) The cells were excited at 540 nm, and 580 nm (dichroic filter) and 590 nm (barrier filter). Measurements were performed and plotted online every 10 s. The experiments were performed at room temperature (22 °C).

# Supplementary Figures

**

**

**Supplementary figure 1. Spontaneous intracellular Ca^2+^ oscillations in C-MSCs.** Spontaneous oscillations in [Ca^2+^]_i_ recorded in a fraction of C-MSCs loaded with the Ca^2+^-sensitive fluorophore, Fura 2/AM. The cells were discarded from subsequent analysis.


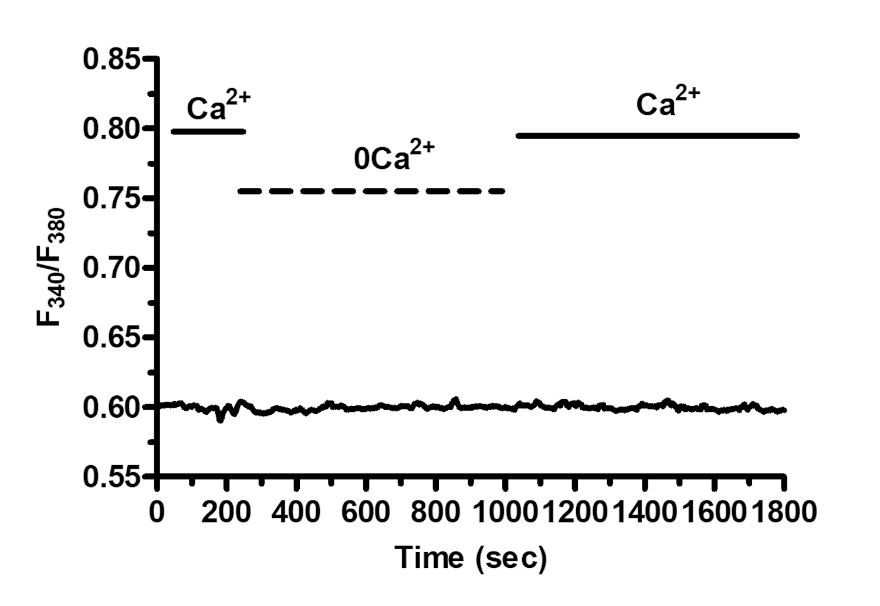


**Supplementary figure 2. Removal of extracellular Ca^2+^ does not activate SOCE in C-MSCs.** Simple removal of extracellular Ca^2+^ does not result in an increase in [Ca^2+^]_i_ upon the subsequent Ca^2+^ restitution to the bath.


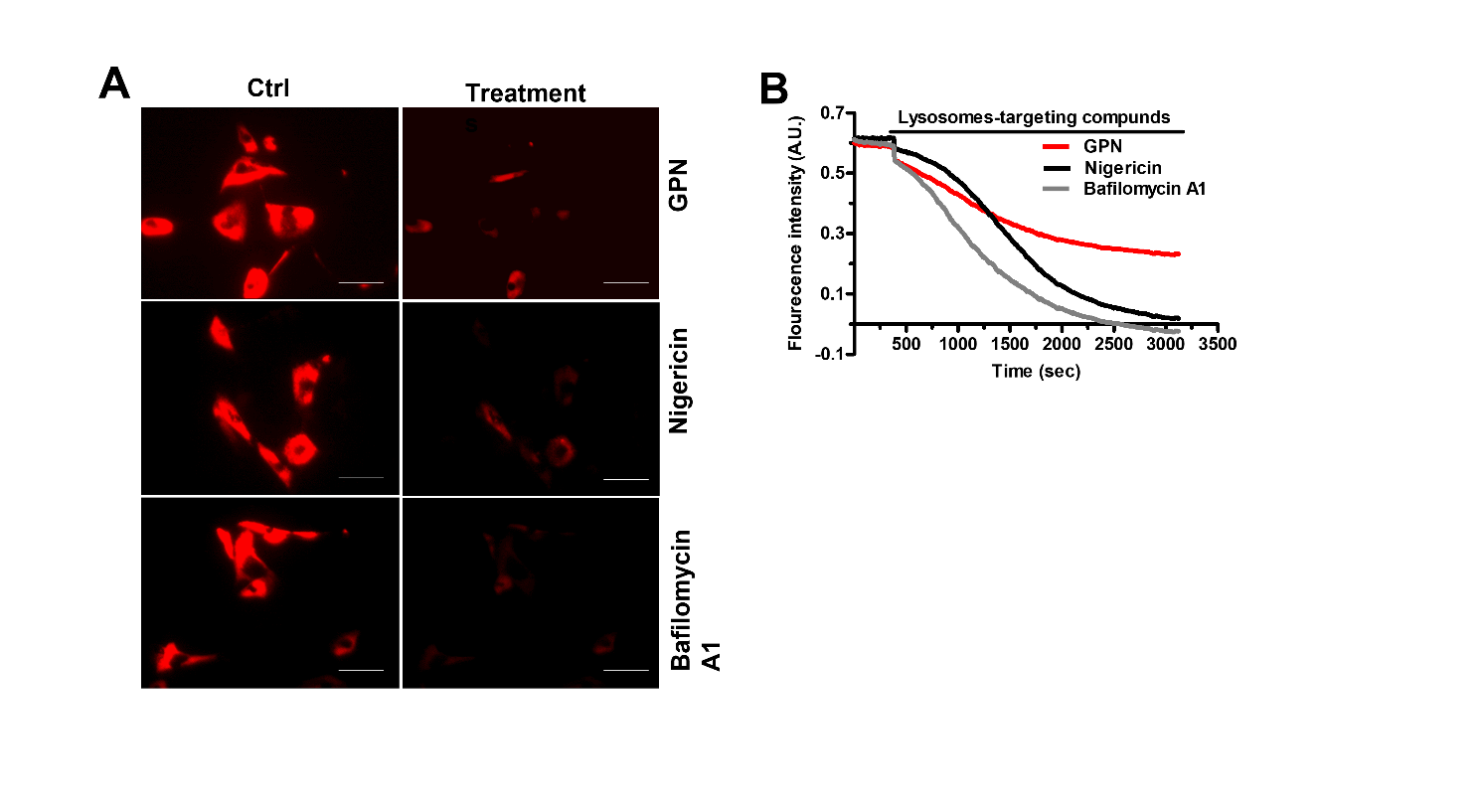


**Supplementary figure 3. Disruption of acidic stores reduced Lysotracker red fluorescence in C-MSCs.** A) C-MSC cells were loaded with Lysotracker red (100 nM, 20 min) before being exposed to GPN (200 µM), nigericin (50 µM) or bafilomycin A1 (1 µM). The images show Lysotracker red fluorescence at the beginning (Ctrl) and at the end of the treatment. The calibration bar is 20 µm. B) Time lapse recording of the changes in Lysotracker red florescence upon application of the lysosomes-targeting compounds.


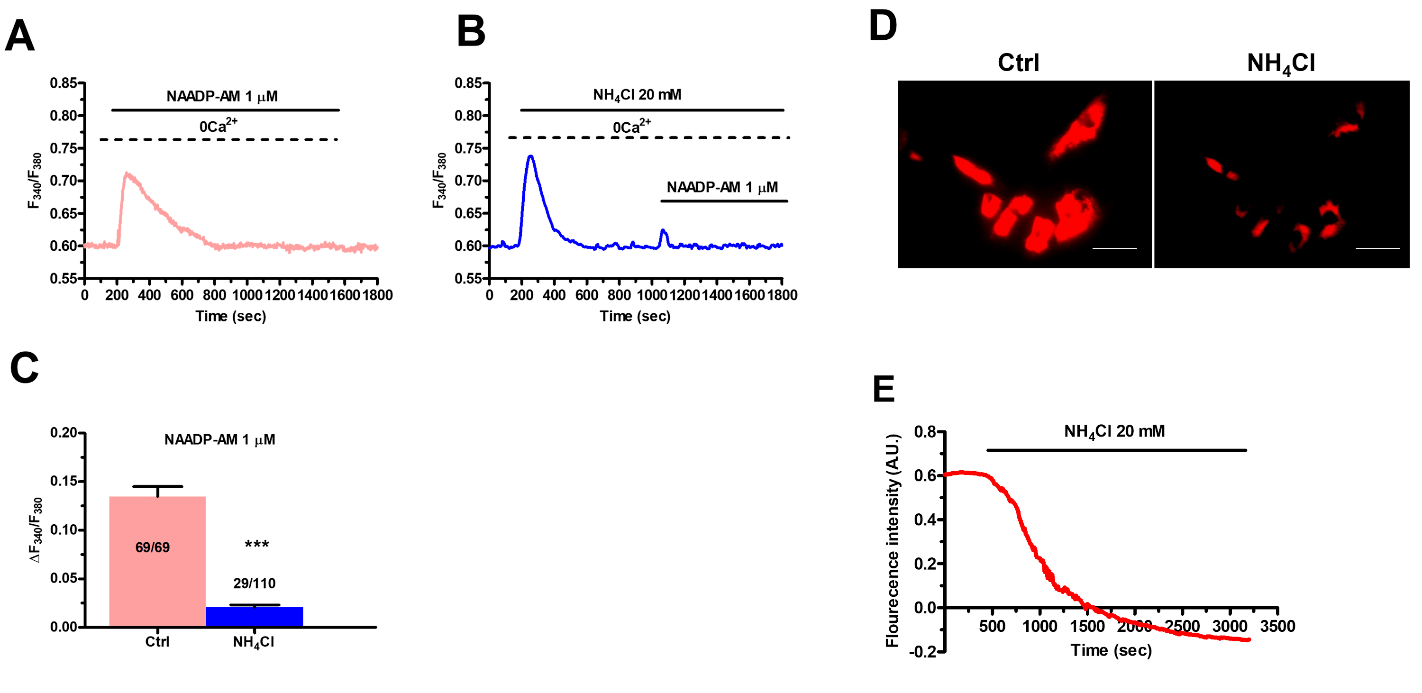


**Supplementary figure 4. NH_4_Cl prevents NAADP-AM-induced intracellular Ca^2+^ mobilization in C-MSCs.** A) NAADP-AM (1 µM) evoke a transient increase in [Ca^2+^]_i_ under 0Ca^2+^ conditions. B) NH_4_Cl (20 mM) evoked a transient elevation in [Ca^2+^]_i_ that prevented the subsequent mobilization of endogenous Ca^2+^ by NAADP-AM. C) mean±SE of the amplitude of NAADP-AM-evoked Ca^2+^ transients in the absence (Ctrl) and presence of NH_4_Cl. Student’s t-test: *** p < 0.001. D) C-MSC cells were loaded with Lysotracker red (100 nM, 20 min) before being exposed to NH_4_Cl (20 mM). The images show Lysotracker red fluorescence at the beginning (Ctrl) and at the end of the treatment. The calibration bar is 20 µm. E) Time lapse recording of the changes in Lysotracker red florescence upon application of NH_4_Cl.





**Supplementary figure 5. InsP_3_Rs and SOCE support nigericin-evoked intracellular Ca^2+^ signals in C-MSCs**. A) Pharmacological depletion of the lysosomal Ca^2+^ store with nigericin (50 µM) induces a transient elevation in [Ca^2+^]_i_ under 0Ca^2+^ conditions (0Ca^2+^) followed by a second increase in [Ca^2+^]_i_ upon Ca^2+^ restitution to the bath. B) Endogenous Ca^2+^ release by nigericin was abrogated by pretreating the cells with 2-APB (50 µM, 30 min), a blocker of InsP_3_Rs, or with U73122 (10 µM, 10 min), an inhibitor of PLC. C) Mean ± SE of the amplitude of the peak Ca^2+^ response to nigericin, under the designated treatments. One-way ANOVA: *** *p* < 0.001.


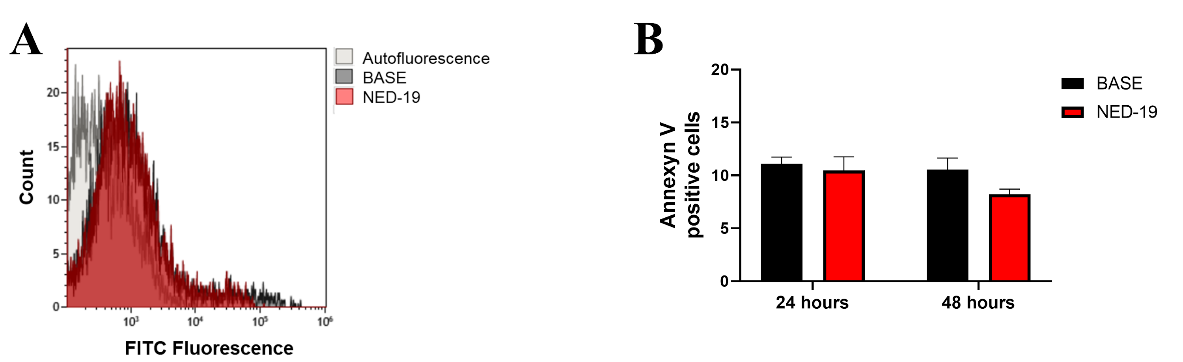


**Supplementary figure 6. NED-19 does not affect C-MSC viability.** Following 4 hours of growth without serum, cardiac mesenchymal stromal cells isolated from healthy control donors were treated with 100 µM NED-19 for 30 minutes and subsequently stimulated with 20% FBS. Following 24 and 48 h of FBS stimulation, the medium was removed, cells detached from the plates, and incubated with Annexin V Dye. A) representative FACS analysis of the FITC emission of unstained C-MSCs (autofluorescence; white), untreated C-MSCs with Annexin V (BASE, gray), and C-MSCs with Annexin V and treated with NED-19 (NED-19, red). B) mean Annexin V emission of HC C-MSC treated or not with NED-19. The results are expressed as mean ± SEM, (n= 3/group).

**References**

[1] F. Moccia, E. Zuccolo, F. Di Nezza, G. Pellavio, P.S. Faris, S. Negri, A. De Luca, U. Laforenza, L. Ambrosone, V. Rosti, G. Guerra, Nicotinic acid adenine dinucleotide phosphate activates two-pore channel TPC1 to mediate lysosomal Ca(2+) release in endothelial colony-forming cells, J. Cell. Physiol., 236 (2021) 688-705.

[2] E. Zuccolo, U. Laforenza, S. Negri, L. Botta, R. Berra-Romani, P. Faris, G. Scarpellino, G. Forcaia, G. Pellavio, G. Sancini, F. Moccia, Muscarinic M5 receptors trigger acetylcholine-induced Ca(2+) signals and nitric oxide release in human brain microvascular endothelial cells, J. Cell. Physiol., 234 (2019) 4540-4562.

[3] A. Spano, S. Barni, V. Bertone, L. Sciola, Changes on lysosomal compartment during PMA-induced differentiation of THP-1 monocytic cells: Influence of type I and type IV collagens, Advances in Bioscience and Biotechnology, 04 (2013) 8-18.

[4] P. Faris, G. Pellavio, F. Ferulli, F. Di Nezza, M. Shekha, D. Lim, M. Maestri, G. Guerra, L. Ambrosone, P. Pedrazzoli, U. Laforenza, D. Montagna, F. Moccia, Nicotinic Acid Adenine Dinucleotide Phosphate (NAADP) Induces Intracellular Ca(2+) Release through the Two-Pore Channel TPC1 in Metastatic Colorectal Cancer Cells, Cancers (Basel), 11 (2019) pii: E542.
